# Coating of a Novel Antimicrobial Nanoparticle with a Macrophage Membrane for the Selective Entry into Infected Macrophages and Killing of Intracellular Staphylococci

**DOI:** 10.1002/adfm.202004942

**Published:** 2020-09-16

**Authors:** Yuanfeng Li, Yong Liu, Yijin Ren, Linzhu Su, Ang Li, Yingli An, Vincent Rotello, Zhanzhan Zhang, Yin Wang, Yang Liu, Sidi Liu, Jian Liu, Jon D. Laman, Linqi Shi, Henny C. van der Mei, Henk J. Busscher

**Affiliations:** State Key Laboratory of Medicinal Chemical Biology, Materials and Ministry, Key Laboratory of Functional Polymer Materials of Ministry of Education, Institute of Polymer Chemistry College of Chemistry, Nankai University, 94 Weijin Road, Tianjin 300071, P. R. China; Department of Biomedical Engineering, University of Groningen and University Medical Center Groningen, Antonius Deusinglaan 1, 9713 AV, Groningen, The Netherlands; Department of Orthodontics, University of Groningen and University Medical Center Groningen, Hanzeplein 1, 9700 RB, Groningen, The Netherlands; State Key Laboratory of Medicinal Chemical Biology, Materials and Ministry, Key Laboratory of Functional Polymer Materials of Ministry of Education, Institute of Polymer Chemistry College of Chemistry, Nankai University, 94 Weijin Road, Tianjin 300071, P. R. China; Department of Biomedical Engineering, University of Groningen and University Medical Center Groningen, Antonius Deusinglaan 1, 9713 AV, Groningen, The Netherlands; State Key Laboratory of Medicinal Chemical Biology, Materials and Ministry, Key Laboratory of Functional Polymer Materials of Ministry of Education, Institute of Polymer Chemistry College of Chemistry, Nankai University, 94 Weijin Road, Tianjin 300071, P. R. China; Department of Chemistry, University of Massachusetts, 710 North Pleasant Street, Amherst, MA 01003, USA; State Key Laboratory of Medicinal Chemical Biology, Materials and Ministry, Key Laboratory of Functional Polymer Materials of Ministry of Education, Institute of Polymer Chemistry College of Chemistry, Nankai University, 94 Weijin Road, Tianjin 300071, P. R. China; Institute of Functional Nano & Soft Materials (FUNSOM), Jiangsu Key Laboratory for Carbon-Based Functional Materials & Devices, Collaborative Innovation Center of Suzhou Nano Science and Technology, Soochow University, 199 Ren’ai Rd, Suzhou, Jiangsu 215123, P. R. China; Department of Biomedical Sciences of Cells and Systems, University of Groningen and University Medical Center Groningen, Antonius Deusinglaan 1, 9713 AV, Groningen, The Netherlands; State Key Laboratory of Medicinal Chemical Biology, Materials and Ministry, Key Laboratory of Functional Polymer Materials of Ministry of Education, Institute of Polymer Chemistry College of Chemistry, Nankai University, 94 Weijin Road, Tianjin 300071, P. R. China; Department of Biomedical Engineering, University of Groningen and University Medical Center Groningen, Antonius Deusinglaan 1, 9713 AV, Groningen, The Netherlands

**Keywords:** bacterial infections, cell membrane encapsulation, ciprofloxacin, Toll-like receptors, triclosan

## Abstract

Internalization of *Staphylococcus aureus* by macrophages can inactivate bacterial killing mechanisms, allowing intracellular residence and dissemination of infection. Concurrently, these staphylococci can evade antibiotics that are frequently unable to pass mammalian cell membranes. A binary, amphiphilic conjugate composed of triclosan and ciprofloxacin is synthesized that self-assemble through micelle formation into antimicrobial nanoparticles (ANPs). These novel ANPs are stabilized through encapsulation in macrophage membranes, providing membrane-encapsulated, antimicrobial-conjugated NPs (Me-ANPs) with similar protein activity, Toll-like receptor expression and negative surface charge as their precursor murine macrophage/human monocyte cell lines. The combination of Toll-like receptors and negative surface charge allows uptake of Me-ANPs by infected macrophages/monocytes through positively charged, lysozyme-rich membrane scars created during staphylococcal engulfment. Me-ANPs are not engulfed by more negatively charged sterile cells possessing less lysozyme at their surface. The Me-ANPs kill staphylococci internalized in macrophages in vitro. Me-ANPs likewise kill staphylococci more effectively than ANPs without membrane-encapsulation or clinically used ciprofloxacin in a mouse peritoneal infection model. Similarly, organ infections in mice created by dissemination of infected macrophages through circulation in the blood are better eradicated by Me-ANPs than by ciprofloxacin. These unique antimicrobial properties of macrophage-monocyte Me-ANPs provide a promising direction for human clinical application to combat persistent infections.

## 1. Introduction

Infection by antimicrobial resistant bacteria is predicted to yield more deaths than cancer, and become the number one cause of death by 2050, due in a large part to the growing number of antibiotic-resistant strains.^[1]^ The challenges generated by this emerging resistance are exacerbated by native mechanisms used by bacteria to evade antimicrobials. One way infectious bacteria evade antibiotic action and killing by host immune cells is by “hiding” in mammalian cells, a pathway that can increase the severity of disease and hampers pathogen eradication.^[2,3]^ The mammalian cell membrane acts as a barrier toward penetration of many antibiotics,^[4]^ which makes the intracellular environment a protective shelter for infecting bacteria. Moreover, the diversity spectrum of enzymes present in host mammalian cells can inactivate antibiotics to further protect bacteria hiding intracellularly. Even macrophages, intended by nature to facilitate bacterial clearance from the body, can provide intracellular shelter to infecting bacteria.^[5]^ In fact, many intracellular bacterial pathogens replicate in the shelter provided by macrophages.^[6]^ After engulfment, bacteria initially reside in membrane-bound phagosomes,^[7]^ that fuse with lysosomes to form phagolysosomes in which bacteria are normally killed by reactive oxygen species, lysozyme and cationic antimicrobial peptides. However, bacteria can remain dormant in the low pH environment of phagosomes as well as reside in phagolysosomes for extended periods of time.^[8–11]^ Moreover, intracellular *Staphylococcus aureus* can delay phagosome fusion with lysozyme for at least 28 h,^[12]^ allowing staphylococci to survive and develop resistance to reactive oxygen species and antimicrobial peptides.^[7,13]^ Apart from bacterial survival, these combined mechanisms allow dissemination of infection through the body by circulation of infected macrophages in the blood.^[14]^ Effective evasion strategies based on hiding in macrophages have been developed by multiple pathogenic bacterial strains, such as *Mycobacterium tuberculosis*, *S. aureus* and *Salmonella enteri*ca.^[15]^ Frequently, extremely high doses of antibiotics are needed to kill intracellular bacteria and prevent their dissemination through circulation in the blood. High dosing can lead to severe side effects for the patients, while being still insufficient to cure infection.^[16,17]^ Overall, eradication of bacteria hiding in macrophages is crucial for the long-term success of antibiotic treatment.^[18,19]^

With the number of antibiotics available to eradicate bacterial infections shrinking at an alarming rate,^[20–22]^ new strategies to eradicate bacteria hiding intracellularly in macrophages and prevent dissemination of infection through the body, are direly needed. Azithromycin and ciprofloxacin can be effectively delivered intracellularly in high amounts when encapsulated in either negatively- or positively charged liposomes.^[23]^ Equipping liposomes with a cell penetrating protein enhanced intracellular delivery of gentamicin,^[15]^ while colistin-loaded liposomes equipped with extracellular adherence proteins from *S. aureus* facilitated entry into HEp-2 and Caco-2 cells and killed intracellular *S. enteri*ca.^[24]^ These liposomes enter mammalian cells through membrane fusion, but their fusogenicity comes at the expense of their stability,^[25]^ and liposomes with increased fusogenicity are more prone to rupture and inadvertent cargo release.^[26,27]^ Synthetic antimicrobial nanoparticles (ANP) can be made more stable by a variety of strategies, but are easily cleared by host immune cells.^[28]^ To avoid clearance by host immune cells and facilitate their transportation through the blood circulation, synthetic antimicrobial nanocarriers can be equipped with stealth properties and pH responsiveness that make them suitable for use in infection control.^[29]^ In general however, synthetic nanocarriers are hard to render biocompatible.

Cell membrane surfaces, by nature meet many of the biocompatibility requirements that are challenging in the design of synthetic nanocarriers.^[30]^ Red blood cell, leukocyte, cancer cell, and platelet membrane-coated nanoparticles have been dubbed as “autogenous friends” with incredibly long blood circulation times to assist tumor therapy.^[31]^ Cell membrane-coated nanoparticles are known for their immune modulating properties and their potential for detoxification.^[32]^ Biomimetic red-blood cell membrane-encapsulated nanoparticles, for instance, have been applied for detoxification of organophosphate poisoning,^[33,34]^ while leukocyte membrane-encapsulated NPs can absorb endo-toxins during sepsis treatment.^[35]^ Encapsulation by leukocyte membranes of NPs also inhibited synovial inflammation and alleviated joint damage.^[36]^ Bacterially stimulated macrophage membrane-coated gold–silver nanocages targeted themselves to bacterial cell surfaces in an extracellular environment.^[37]^ The use of bacterially pretreated macrophage membranes for encapsulation of nanoparticles (NPs) is highly unpractical however, and to our knowledge native macrophage membrane-encapsulated NPs have not yet been applied to cure intracellular bacterial infections that are even more difficult to treat than extracellular infections and form a cause of recurrence. Thus, inspired by the natural biocompatibility of unstimulated macrophages, we encapsulated ANPs in membranes from J774A.1 (as a model for mouse macrophages) and THP-1 (as a model for human monocytes) to provide membrane-encapsulated, antimicrobial NPs (Me-ANPs) that take advantage of the biocompatibility, resistance to clearance by host immune cells, and bacterial pathogen-targeting ability that macrophages possess by nature.^[30]^

**Figure 1.**
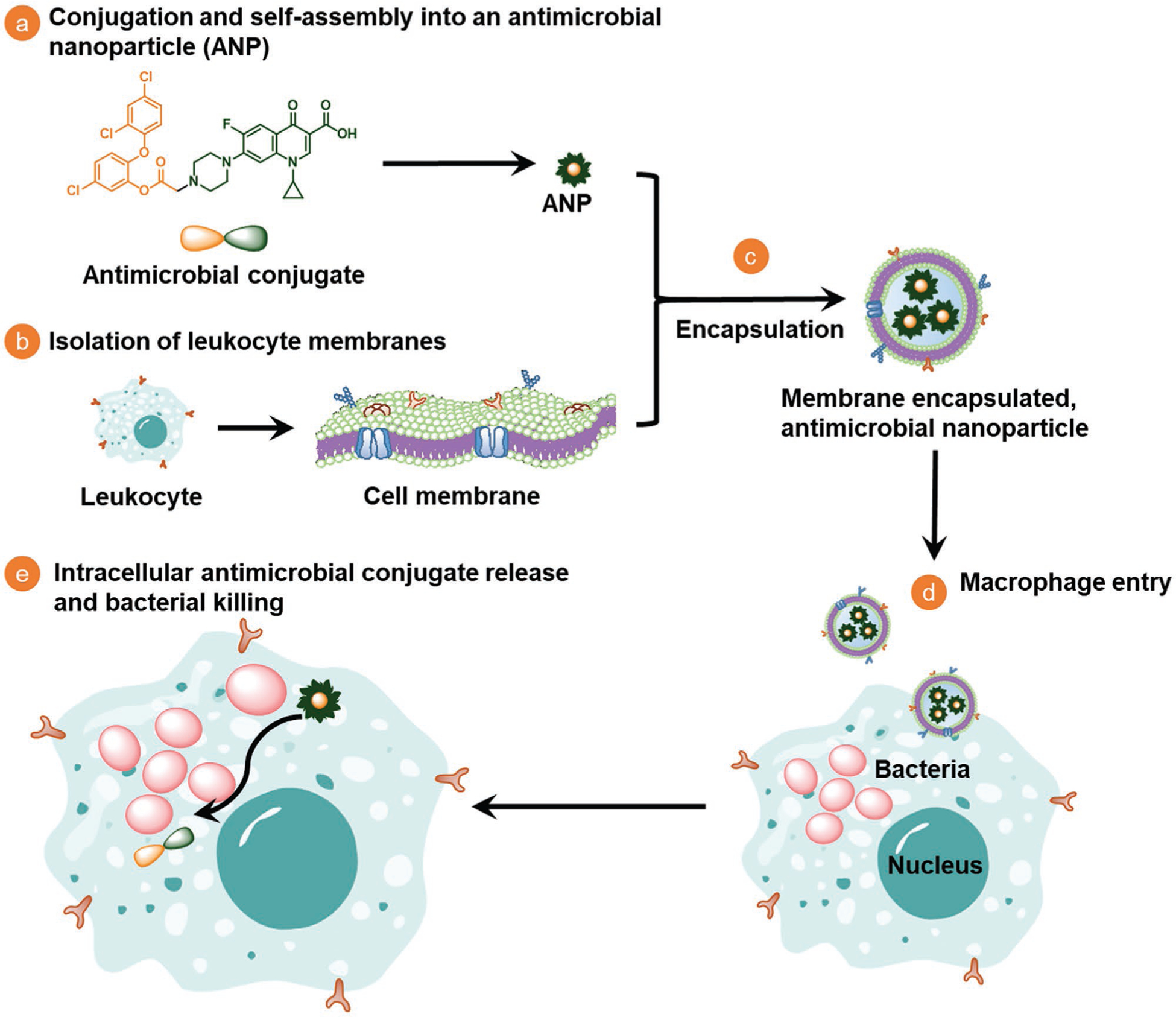
Design, synthesis and hypothesized mechanism of macrophage-monocyte membrane-encapsulated, antimicrobial-conjugated nanoparticles (Me-ANPs) to kill intracellular bacterial pathogens, hiding inside leukocytes. a) Preparation of a hydrophobic antimicrobial (triclosan) and a more hydrophilic antibiotic (ciprofloxacin) into an amphiphilic, binary antimicrobial-conjugate that self-assembles through micelle formation in aqueous solution to form antimicrobial NPs. b) Isolation of leukocyte membranes. c) Stabilization of self-assembled, antimicrobial NPs by encapsulation in leukocyte membranes. d) Macrophage-monocytes Me-ANPs are naturally engulfed into an infected macrophage. e) Once inside an infected macrophage, intracellular release of conjugated antimicrobials from Me-ANPs kills infecting bacteria. Details not drawn to scale.

The novel ANPs that provide the foundation of our system were composed of amphiphilic, binary antimicrobial-conjugates composed of a hydrophobic antimicrobial, triclosan, and a common clinically applied, more hydrophilic antibiotic, ciprofloxacin (Figure 1). Triclosan was chosen as an antimicrobial, because it is not only a common oral antimicrobial in tooth-pastes and mouthwashes,^[38]^ but also applied in antimicrobial sutures^[39]^ and other clinical infection control measures.^[40]^ The amphiphilic antimicrobial-conjugates self-assembled into ANPs that were subsequently stabilized by macrophage-monocyte membrane-encapsulation. The killing efficacy of Me-ANPs toward bacteria hiding in macrophages was demonstrated both in vitro and in vivo, using *S. aureus* as an infecting organism. *S. aureus* is a leading cause of severe bacterial infections world-wide and currently regarded as one of the most prevalent intracellular human pathogen.^[41]^ *S. aureus* is hard to kill once intracellularly present.^[42,43]^ Both in a mouse peritoneal infection model as well as in a mouse organ infection model, infection was more effectively eradicated by macrophage membrane-encapsulated Me-ANPs than by ANPs without membrane-encapsulation or ciprofloxacin, a common antibiotic in clinical practice.

## 2. Results

### 2.1. Preparation and Characterization of Macrophage-Monocyte Membrane-Encapsulated, Antimicrobial-Conjugated Nanoparticles

**Figure 2.**
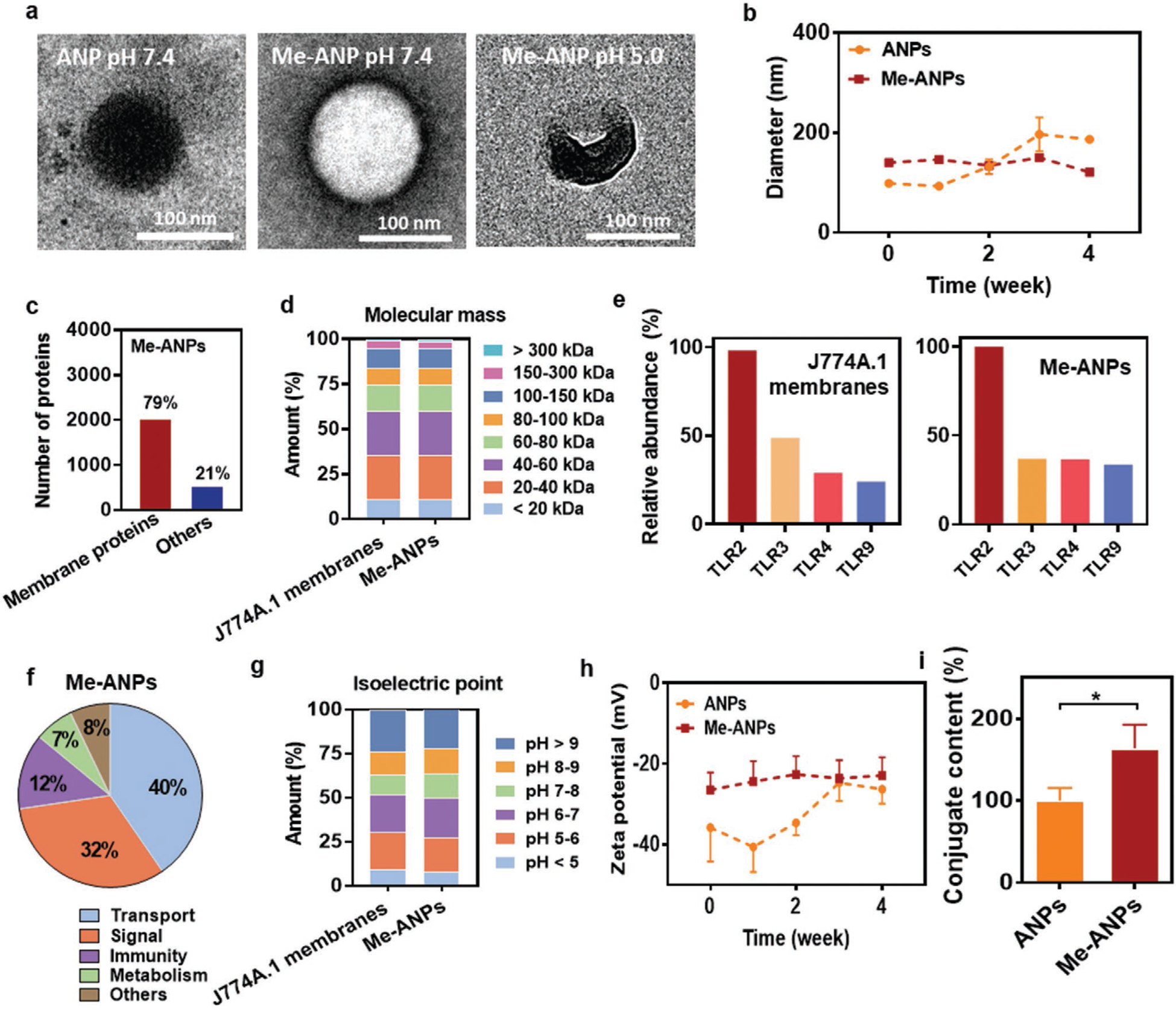
Characteristics of mouse J774A.1 membrane-encapsulated, antimicrobial-conjugated nanoparticles (Me-ANPs). a) Transmission electron micrographs of a negative-stained (0.5% uranyl acetate) ANP and a Me-ANP after 24 h in 10 × 10^−3^ M potassium phosphate buffer at different pH, showing membrane-encapsulation as a dark area around the NP. Note partial disassembly of the Me-ANP at pH 5.0. Lower magnification electron micrographs showing more ANPs are presented in Figure S5 (Supporting Information). b) Hydrodynamic diameters in 10 × 10^−3^ M potassium phosphate buffer of ANPs in absence and presence of membrane-encapsulation, as a function of storage time in buffer. Data were expressed as means ± standard deviations over triplicate nanocarrier preparations. c) The number of J774A.1 membrane proteins and other proteins on Me-ANP identified by liquid chromatography-mass spectrometry (LC-MS). d) Molecular mass (kDA) distribution of membrane proteins in mouse J774A.1 membranes and Me-ANPs by LC-MS. e) Relative abundance of Toll-like receptors identified using LC-MS involved in bacterial recognition on J774A.1 membranes and Me-ANPs. and abundance of TLR-2 in J774A.1 membranes set at 100%. f) Functional characterization of membrane proteins identified on Me-ANPs. Proteins were classified according to UniProt/GO database^[51]^ using the LC-MS data as input. g) Same as panel (d), now for the distribution of isoelectric points of membrane proteins. h) Zeta potentials in 10 × 10^−3^ M potassium phosphate buffer of ANPs in absence and presence of membrane-encapsulation, as a function of storage time in buffer. Data were expressed as means ± standard deviations over triplicate nanocarrier preparations. i) Antimicrobial-conjugate content in wt% of Me-ANPs, expressed relative to the initial antimicrobial-conjugate content of ANPs in absence of encapsulation. Antimicrobial-conjugate contents were derived from UV–vis spectroscopy (see Figure S6, Supporting Information). Data are expressed as means ± standard deviations over triplicate nanocarrier preparations. Asterisks above the data points indicate statistical significance at *p* < 0.05 (*, Student’s *t*-test) between ANPs and Me-ANPs.

Amphiphilic, binary antimicrobial-conjugates were synthesized via chloroacetylation of triclosan and subsequent chloride substitution using ciprofloxacin with an overall yield of 70%. The antimicrobial-conjugate composition was confirmed by ^1^H and ^13^C NMR (Figures S1 and S2, respectively, Supporting information) and electrospray ionization mass spectrometry (Figure S3, Supporting Information). Conjugation was done through reacting the hydroxyl group of triclosan and the secondary amine group of ciprofloxacin. These groups were selected because esterification of the phenol group of triclosan and alkylation of the piperazinyl group of ciprofloxacin have been demonstrated to have little negative effect on antimicrobial efficacy.^[44,45]^ The critical micelle concentration of the antimicrobial conjugate was 0.38 μg mL^−1^ (Figure S4, Supporting Information). For self-assembly of the amphiphilic antimicrobial-conjugate through micelle formation into an antimicrobial NP-template, conjugates were dissolved in dimethylsulfoxide, added dropwise into water and centrifuged to remove the organic solvent.^[46]^ Next, membranes isolated from mouse J774A.1 cells were mixed with the NPs to obtain J774A.1 Me-ANPs. Electron microscopy showed spherically-shaped ANPs and Me-ANPs after 24 h in a 10 × 10^−3^ m potassium phosphate buffer at pH 7.4, with the encapsulating membrane clearly visible (Figure 2a). Me-ANPs partly disassembled after 24 h exposure to pH 5.0, but not at the physiological pH of 7.4 (Figure 2a). Freshly made ANPs had diameters of around 90 nm (Figure 2b) with a polydispersity index (PDI) of 0.11 in absence of J774A.1 membrane-encapsulation. After 2 weeks storage in potassium phosphate buffer at pH 7.4, ANP diameters increased roughly twofold, indicative of the water uptake. Membrane-encapsulation yielded an increase in diameter to around 110 nm and in PDI to 0.13. Moreover, membrane encapsulation stabilized the Me-ANP diameter up to at least 4 weeks after preparation. Liquid chromatography-mass spectrometry (LC-MS) analysis^[28,47]^ indicated that 79% of the proteins found on J774A.1 Me-ANPs were membrane proteins (Figure 2c), as confirmed by the similarity in molecular mass distributions in J774A.1 membranes and Me-ANPs (Figure 2d). Toll-like receptors involved in recognition of bacterial lipopolysaccharides and lipoteichoic acids, i.e., cell membrane derived TLR2 and TLR4 as well as endosomal membrane-derived TLR3 and TLR9,^[48–50]^ were also abundantly present both on J774A.1 membranes and Me-ANPs (Figure 2e). A functional classification based on UniProt/GO^[51]^ analysis using the LC-MS data as input, identified the membrane proteins on Me-ANPs to be involved in transport (40%), signaling (32%), immunity (12%) and metabolism (7%) (Figure 2f). This functional classification is in line with protein functions in J774A.1 membranes.^[52]^ Actual protein activity is virtually impossible to measure in a mixture of over 4000 proteins identified in a macrophage membrane, but absence of denaturation can be anticipated based on absence of aggregation and precipitation (common to denatured proteins) of Me-ANPs over a period of four weeks. This makes maintenance of functional protein activity likely. Based on isoelectric points (pI; Figure 2g), the percentages of positively (pI > 7) and negatively (pI < 7) charged proteins were equal for J774A.1 membranes and Me-ANPs. Few (<9%) highly negatively charged proteins (pI < 5) were identified, while more than 20% of the proteins carried a high positive charge (pI > 9). Accordingly, J774A.1 membrane-encapsulation compensated the highly negative zeta potential of ANPs in absence of a membrane coating, yielding stable, negative zeta potentials of around −25 mV over the course of at least 4 weeks (Figure 2h). Using UV–vis absorption spectroscopy and setting the antimicrobial-conjugate content of ANPs in absence of encapsulation at 100% (see Figure S6, Supporting Information), additional antimicrobial-conjugates were found to be captured during the encapsulation process (Figure 2i). Thus, membrane-encapsulation of ANPs not only stabilized the particles, but also increased their antimicrobial-conjugate content.

**Figure 3.**
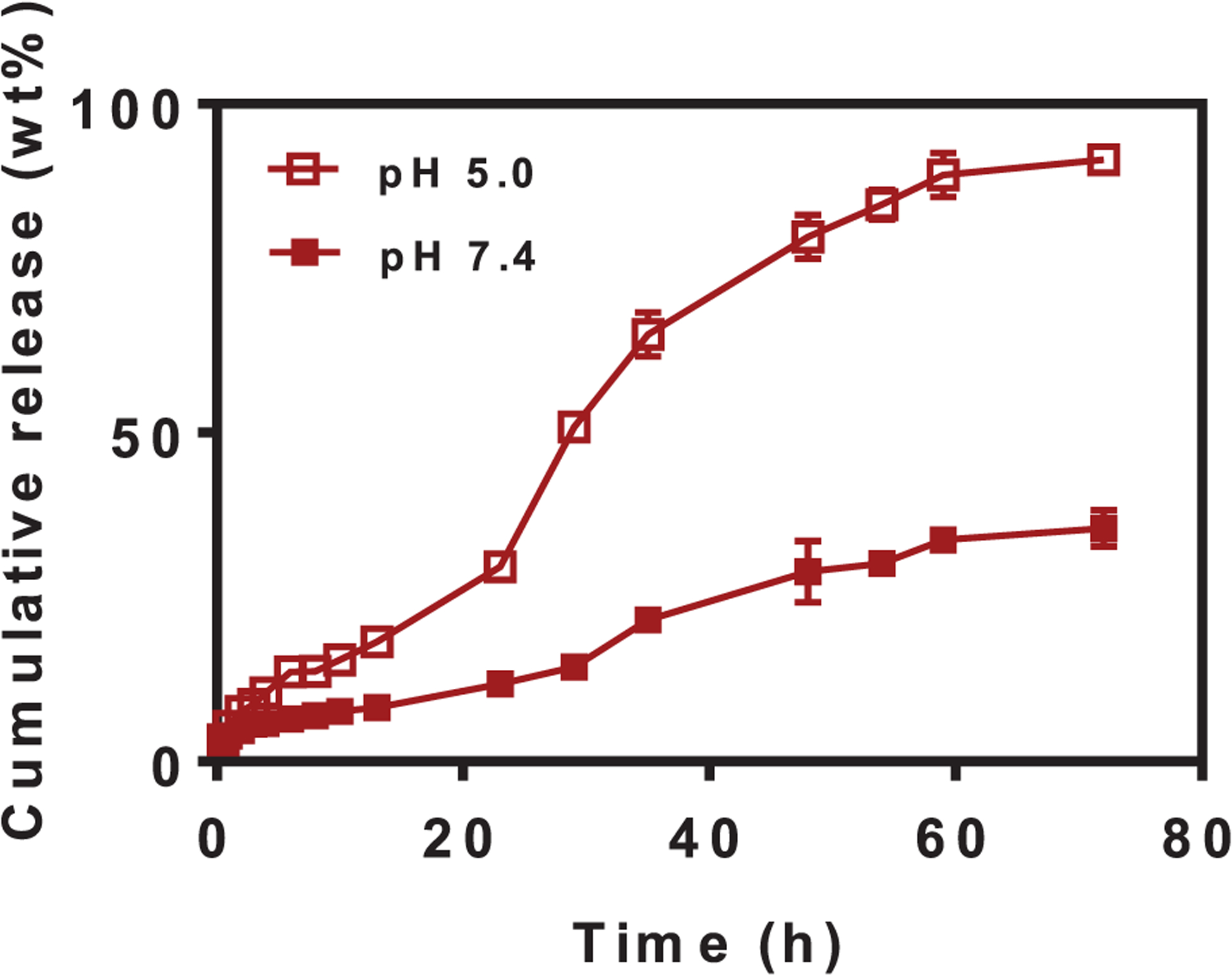
Cumulative antimicrobial-conjugate release from mouse J774A.1 membrane-encapsulated antimicrobial-conjugated nanoparticles as a function of exposure time to phosphate buffered saline (PBS: 10 × 10^−3^ m potassium phosphate with 150 × 10^−3^ m NaCl added) at pH 5.0 and 7.4. The discontinuity in the release curve at pH 5.0 at around 24 h is likely related to disassembly of the nanoparticles (compare Figure 2a). Drug release was measured using UV–vis spectroscopy. Data are expressed as means ± standard deviations over triplicate nanocarrier preparations.

Exposure of J774A.1 Me-ANPs to phosphate buffered saline (PBS) at pH 7.4 only yielded partial release of <50 wt% of the antimicrobial-conjugates over 72 h (Figure 3), but at a lower pH, as prevalent in macrophage sub-cellular compartments,^[53]^ nearly 100% of the antimicrobial-conjugates was released. This enhanced release at low pH, particularly increasing after 24 h, is due to the pH dependent disassembly of Me-ANPs, occurring only in a low pH environment (see also Figure 2a), which is an advantageous feature yielding enhanced availability of the antimicrobials in the same sub-cellular compartments as in which also bacteria reside. Note from Figures 2a and 3, that this disassembly is a slow process occurring over a time scale of around 24 h. Disassembly of Me-ANPs in a low pH environment (pH 5.0) likely arises through protonation of three tertiary amine groups on the antimicrobial conjugate (see Figure 1), possessing p*K*a’s around 4.85 (ChemDraw calculation). Protonation of the amine groups increases the hydrophilicity of the conjugate, which disrupts the balance between its hydrophobic and hydrophilic part that provides the basis for macrophage membrane-stabilized ANP formation.

In a separate series of experiments, Figure S7a–h (Supporting Information) show that encapsulation of ANPs by human THP-1 cells, modeling monocytes, yielded similar stabilization of the nanoparticle diameters and other important properties, including antimicrobial-conjugate release and membrane protein function in the coating as encapsulation by mouse J774A.1 cells.

### 2.2. Engulfment of Macrophage-Monocyte Membrane-Encapsulated, Antimicrobial-Conjugated Nanoparticles into Macrophages In Vitro

**Figure 4.**
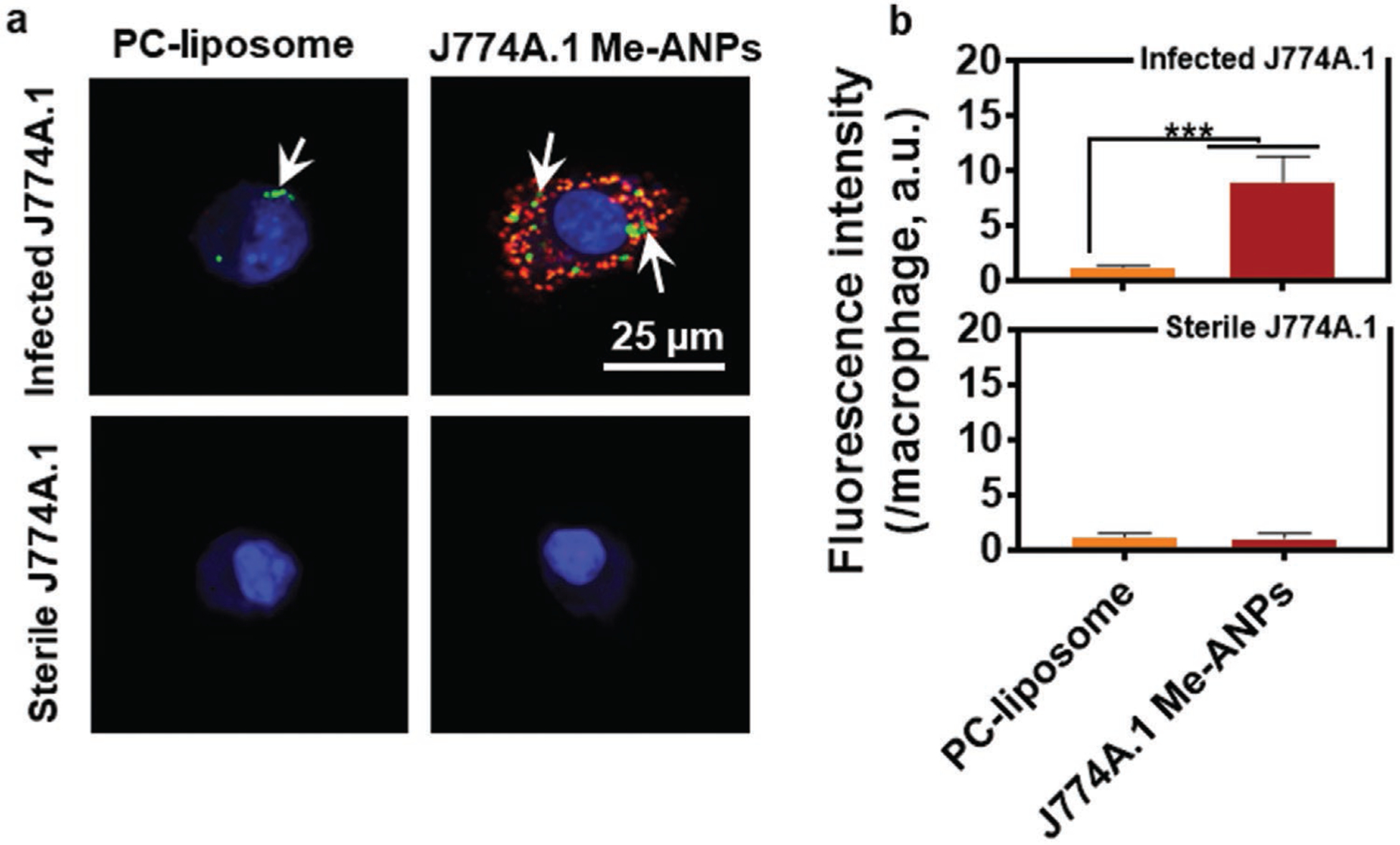
Engulfment of mouse J774A.1 membrane-encapsulated, antimicrobial-conjugated nanoparticles by infected and sterile J774A.1 macrophages with and without in vitro internalized green-fluorescent *S. aureus* WH^GFP^, respectively (2 h exposure time). Experiments were done in PBS. ANPs encapsulated in phosphocholine liposomes (PC-liposomes) are included as a negative control. a) CLSM images illustrating intracellular presence of green-fluorescent staphylococci (arrows) with attached Nile red-loaded, membrane or liposome encapsulated ANPs into J774A.1 macrophages with or without intracellular *S. aureus*. J774A.1 nuclei were blue-fluorescently stained using DAPI. Lower magnification CLSM images showing more macrophages are presented in Figure S8 (Supporting Information). b) Red-fluorescence intensity of Nile red-loaded, membrane or liposome encapsulated ANPs after engulfment in J774A.1 macrophages with or without intracellular *S. aureus*. Data were expressed as a mean fluorescence intensity per macrophage ± standard deviations over triplicate nanocarrier preparations. Three images were blindly chosen in each experiment, comprising on average ≈15 macrophages per image.

To demonstrate engulfment of J774A.1 Me-ANPs into sterile and infected J774A.1 cells, Me-ANPs were loaded with hydrophobic fluorescent Nile red. To obtain infected J774A.1 cells with in vitro internalized *S. aureus*, overnight cultures of staphylococci and cells were grown to internalize staphylococci inside the macrophages, while washing off extracellular bacteria and killing possible remaining extra-cellular staphylococci by exposure to gentamicin.^[16,54,55]^ Intracellular presence of staphylococci was clearly indicated in CLSM images of green-fluorescent staphylococci inside J774A.1 macrophages (see Figure 4a).

Next, J774A.1 macrophages with or without intracellular green-fluorescent *S. aureus* WH^GFP^ were exposed to suspensions of Nile red-loaded, Me-ANPs in PBS and imaged using CLSM. Surprisingly, Me-ANPs can be seen to be exclusively engulfed by infected mouse J774A.1 macrophages with intracellular staphylococci, but not microscopically visible by sterile J774A.1 cells (see also Figure 4a). Similarly, no engulfment was observed of Nile red-loaded ANPs encapsulated in negatively-charged (zeta potential −10 mV)^[56]^ phosphocholine liposomes,^[52]^ both for J774A.1 cells without and with intracellular staphylococci. The above conclusions drawn from Figure 4a were supported by quantitative analyses of the red-fluorescence intensity per macrophage averaged over multiple nanocarrier preparations and macrophages as presented in Figure 4b. However, a very small fluorescence intensity was measured for sterile J774A.1 cells compared to infected macrophages, indicating minor engulfment of Me-ANPs by sterile J774A.1. Me-ANPs encapsulated by THP-1 cells as a model cell line for human monocytes, yielded the same, selective engulfment into infected J774A.1 cells, as exhibited by mouse J774A.1 Me-ANPs (Figure S7i, Supporting Information).

### 2.3. Selectivity of Me-ANPs towards Infected versus Sterile Macrophages

In order to find differences between sterile and infected macrophages relevant for the selectivity of membrane-encapsulated ANPs towards infected macrophages, we compared the zeta potentials of J774A.1 membrane fragments from sterile and infected cells in 10 × 10^−3^ m potassium phosphate buffer.

**Figure 5.**
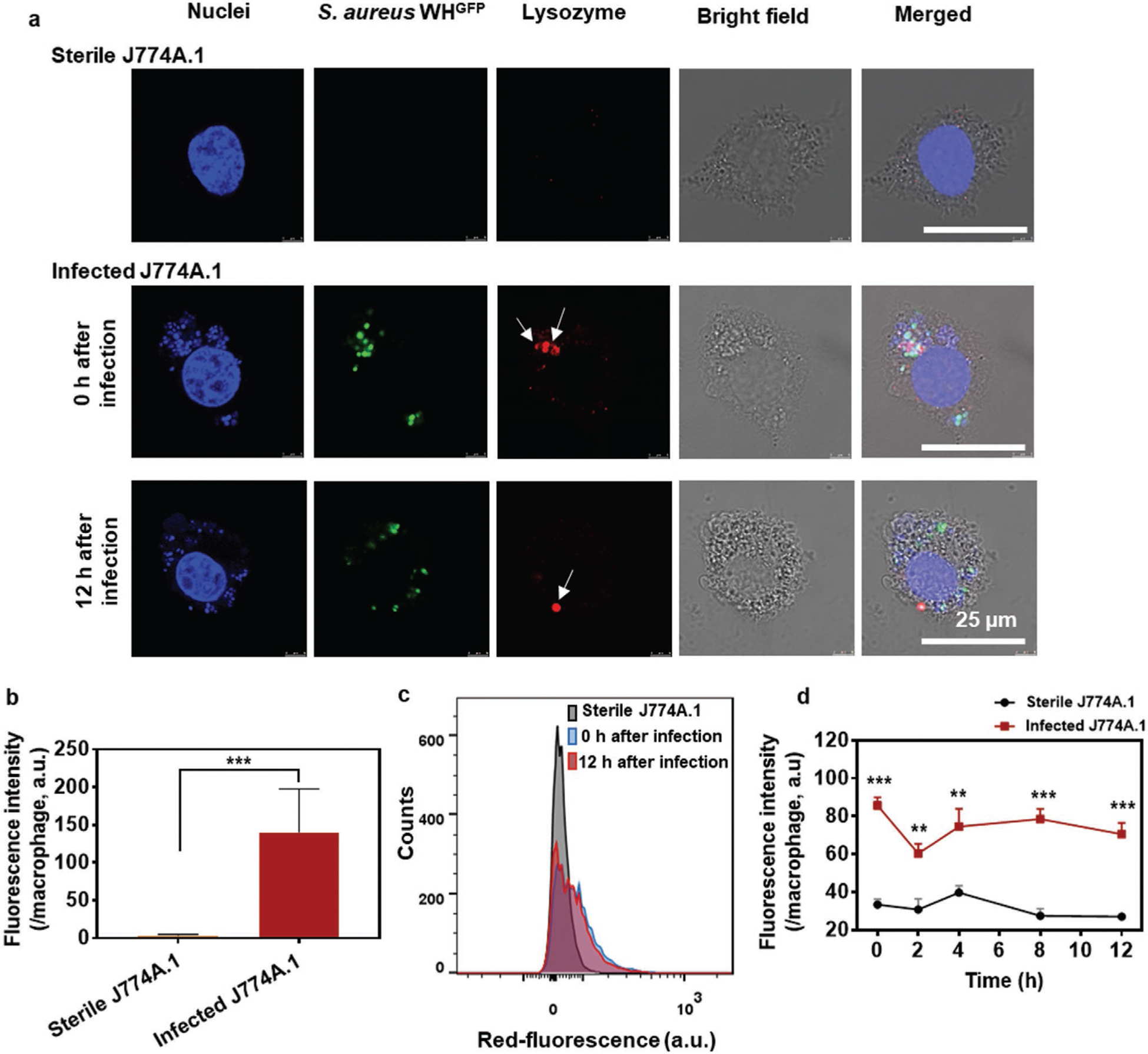
Lysozyme presence on sterile and infected J774A.1 cells. Red-fluorescent anti-lysozyme antibodies were applied for labeling in absence of Triton X-100, labeling only lysozyme on the cell membrane.^[59]^ a) Demonstration of red-fluorescent, lysozyme-rich scars in mouse J774A.1 cells after infection with green-fluorescent *S. aureus* WH^GFP^, including absence of lysozyme-rich scars on sterile cells using CLSM. Blue-fluorescence is due to DAPI staining of macrophage nuclei. Arrows point to a lysozyme-rich spot, coinciding with the likely engulfment-point of staphylococci in an infected cell. Lower magnification CLSM images are presented in Figure S10 (Supporting Information). b) Red-fluorescence intensity due to lysozyme on sterile and infected J774A.1 cells immediately after infection, measured from the intensity in CLSM images. Data were expressed as a mean fluorescence intensity per macrophage ± standard deviations over triplicate nanocarrier preparations. Three images were blindly chosen in each experiment, comprising on average ≈5 macrophages per image. Asterisks above the data points indicate statistical significance at *p* < 0.001 (***). c) Red-fluorescence counts on sterile J774A.1 and infected J774A.1 cells immediately (0 h) and 12 h after infection, measured using flow cytometry. d) Red-fluorescence intensity of lysozyme on sterile J774A.1 cells and infected J774A.1 cells as a function of time after infection. Data were expressed as a mean fluorescence intensity per macrophage ± standard deviations over triplicate nanocarrier preparations. Three images were blindly chosen in each experiment, comprising on average approximately 5 macrophages per image. Asterisks above the data points indicate statistical significance at *p* < 0.001 (***), *p* < 0.01 (**), Student’s *t*-test between sterile and infected cells at each time point.

Membrane fragments from sterile cells had a significantly more negative zeta potential (−47 ± 3 mV), than Me-ANPs (see Figure 2h). Membrane fragments of infected cells, were 10 mV less negatively-charged (zeta potential −37 ± 3 mV) than when isolated from sterile cells. The less negative zeta potentials of cell membranes after staphylococcal infection, suggests capture of positively charged antimicrobial proteins in the membrane during staphylococcal engulfment. For this, lysozyme is a clear candidate protein, as it is a positively charged antimicrobial used by leukocytes for intra- and extraleukocyte degradation of bacterial cell wall peptidoglycans, hydrolyzation the glycan backbone, and killing bacteria through its cationic features.^[57,58]^ Fluorescence labeling with anti-lysozyme antibodies of infected mouse J774A.1 cells demonstrated small lysozyme-rich spots, coinciding with the likely (see also Figure S9, Supporting Information) point of staphylococcal engulfment, that were absent on sterile cells (Figure 5a). Quantification of red-fluorescence intensity of lysozyme in CLSM images was found significantly higher (*p* < 0.001, Student’s t-test) in infected J774A.1 cells than in sterile ones (Figure 5b), in line with the less negative zeta potential of infected J774A.1 cells. These positively-charged, lysozyme scars present the likely point of entry of negatively-charged Me-ANPs as a result of the electrostatic double-layer interaction between opposite charges. Human THP-1 cells immediately after infection demonstrated similar lysozyme scars and overall higher lysozyme presence as observed for infected mouse J774A.1 cells (compare Figure 5 and Figure S11, Supporting Information). Considering the possibility that lysozyme concentrated in the scar-region diffuses to equally distribute over the cell membrane, lysozyme scars on J774A.1 cells were monitored as a function of time after infection. Lysozymes scars remained present over at least 12 h after infection in CLSM images (see also Figure 5a). This result is supported by flow cytometry (Figure 5c), indicating that the lysozyme levels on infected cells were significantly above the lysozyme levels on sterile cells at all points in time up to minimally 12 h (Figure 5d). Thus, the window of opportunity for selective entry of Me-ANPs into infected macrophages is at minimum 12 h.

### 2.4. Antimicrobial Efficacy of Macrophage-Monocyte Membrane-Encapsulated, Antimicrobial-Conjugated Nanoparticles In Vitro

**Figure 6.**
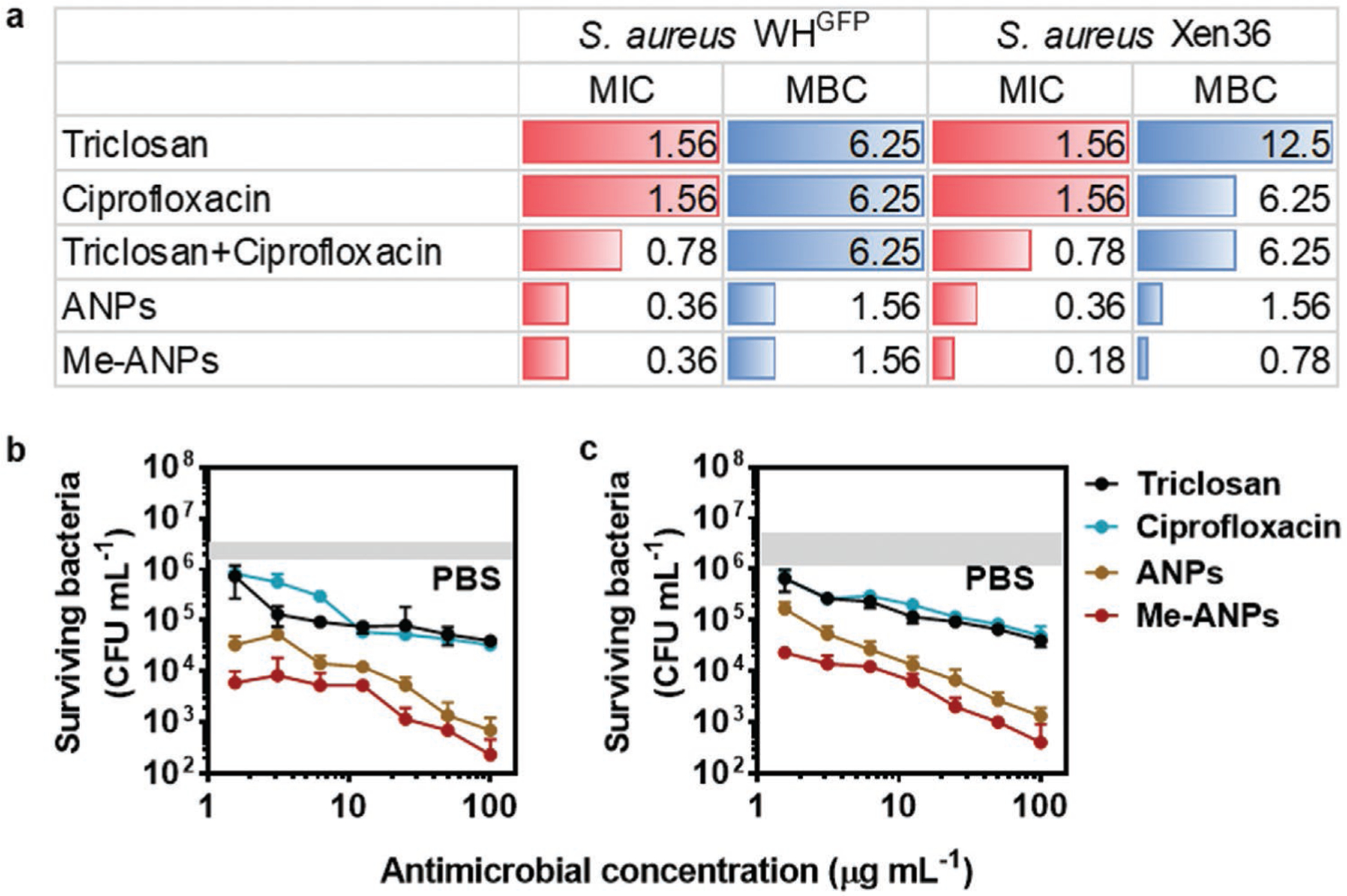
In vitro antimicrobial efficacy of mouse J774A.1 membrane-encapsulated, antimicrobial-conjugated nanoparticles (Me-ANPs), as compared with the composing antimicrobials in solution. a) Minimal inhibitory and bactericidal concentrations (MIC and MBC, respectively) in μg mL^−1^ of planktonic *S. aureus* WH^GFP^ and *S. aureus* Xen36 in suspension for triclosan, ciprofloxacin, their combination, and ANPs without and with J774A.1 membrane-encapsulation in PBS at pH 7.4. Experiments were done at equal concentrations of triclosan and ciprofloxacin. b) Colony forming units of surviving *S. aureus* WH^GFP^ inside J774A.1 cells with in vitro internalized staphylococci after 24 h exposure to triclosan, ciprofloxacin in solution or ANPs with or without J774A.1 membrane-encapsulation. Data were represented by means ± standard deviations over triplicate experiments with independent bacterial cultures. The grey band indicates the initial numbers of CFU mL^−1^ in PBS. c) Same as panel (b), now for *S. aureus* Xen36.

First, to establish whether antimicrobial conjugation and leukocyte membrane-encapsulation had affected the antimicrobial efficacy of the composing antimicrobials, their minimal inhibitory and bactericidal concentrations (MIC and MBC, respectively) towards two multidrug resistant^[22]^ *S. aureus* strains were compared with those of triclosan or ciprofloxacin in solution or a solution with equal concentrations of both antimicrobials. MICs and MBCs of a solution with equal concentrations of triclosan and ciprofloxacin were one serial dilution lower for MIC than of solutions with only triclosan or ciprofloxacin, which is a microbiologically negligible difference. MBC values were likewise similar (Figure 6a). J774A.1 Me-ANPs had twofold lower MIC and fourfold lower MBC than corresponding solutions of both antimicrobials. Thus, it can be conservatively concluded, that neither conjugation nor membrane-encapsulation negatively affected antimicrobial efficacy, pointing to a correct choice of the triclosan and ciprofloxacin sites sacrificed for conjugation. Human THP-1 monocyte membrane-encapsulation yielded identical MIC and MBC for both strains, as did mouse J774A.1 macrophage-membrane encapsulation (Figure S7j, Supporting Information).

Next, J774A.1 macrophages with in vitro internalized staphylococci were exposed to different concentrations of either of the two antimicrobials, or J774A.1 Me-ANPs, while using PBS (pH 7.4) as a negative control. Me-ANPs demonstrated two to three log-units better killing of intracellular staphylococci than triclosan, ciprofloxacin, or PBS (Figure 6b,c), both for internalized *S. aureus* WH^GFP^ (Figure 6b) and internalized *S. aureus* Xen36 (Figure 6c). Importantly, Figure 6b,c indicate that triclosan and ciprofloxacin killed fewer intracellular staphylococci than ANPs in the absence of J774A.1 membrane encapsulation, while ANPs without membrane-encapsulation killed bacteria less effectively than of membrane-encapsulated ones. Hence, the effects of the ANPs themselves and their membrane-encapsulation can be delineated, showing clearly the importance of both self-assembly of the antimicrobial-conjugates into an antimicrobial nanoparticle and their membrane encapsulation (see Figure 1).

### 2.5. In Vitro Cytotoxicity of Macrophage-Monocyte Membrane-Encapsulated, Antimicrobial-Conjugated Nanoparticles

Absence of cytotoxicity of Me-ANPs in vitro was demonstrated based on a metabolic activity assay applied for a variety of cell lines, including human skin fibroblasts, human epithelial colorectal adenocarcinoma cells, murine macrophages and human monocytes (Figure S12, Supporting Information). Metabolic activity of neither cell line was negatively affected by growth in presence of Me-ANPs.

### 2.6. In Vivo Internalization of Staphylococci in Mouse Macrophages

A peritoneal infection model^[60]^ provides a relatively easy tool (see schematics in Figure S13a, Supporting Information) to isolate peritoneal macrophages with in vivo internalized, intracellular staphylococci.^[61]^ To this end, staphylococci were injected in the peritoneal cavity of eight week-old female ICR (CD-1) mice. To obtain infected macrophages with in vivo internalized staphylococci, peritoneal fluid extract was taken 1 d after staphylococcal injection, harvesting 5×10^7^ macrophages mL^−1^ with on average 8 internalized staphylococci per cell (Figure S13b,c, Supporting Information).

### 2.7. Eradication of Peritoneal Infection in Mice Using Macrophage Membrane-Encapsulated, Antimicrobial-Conjugated Nanoparticles

**Figure 7.**
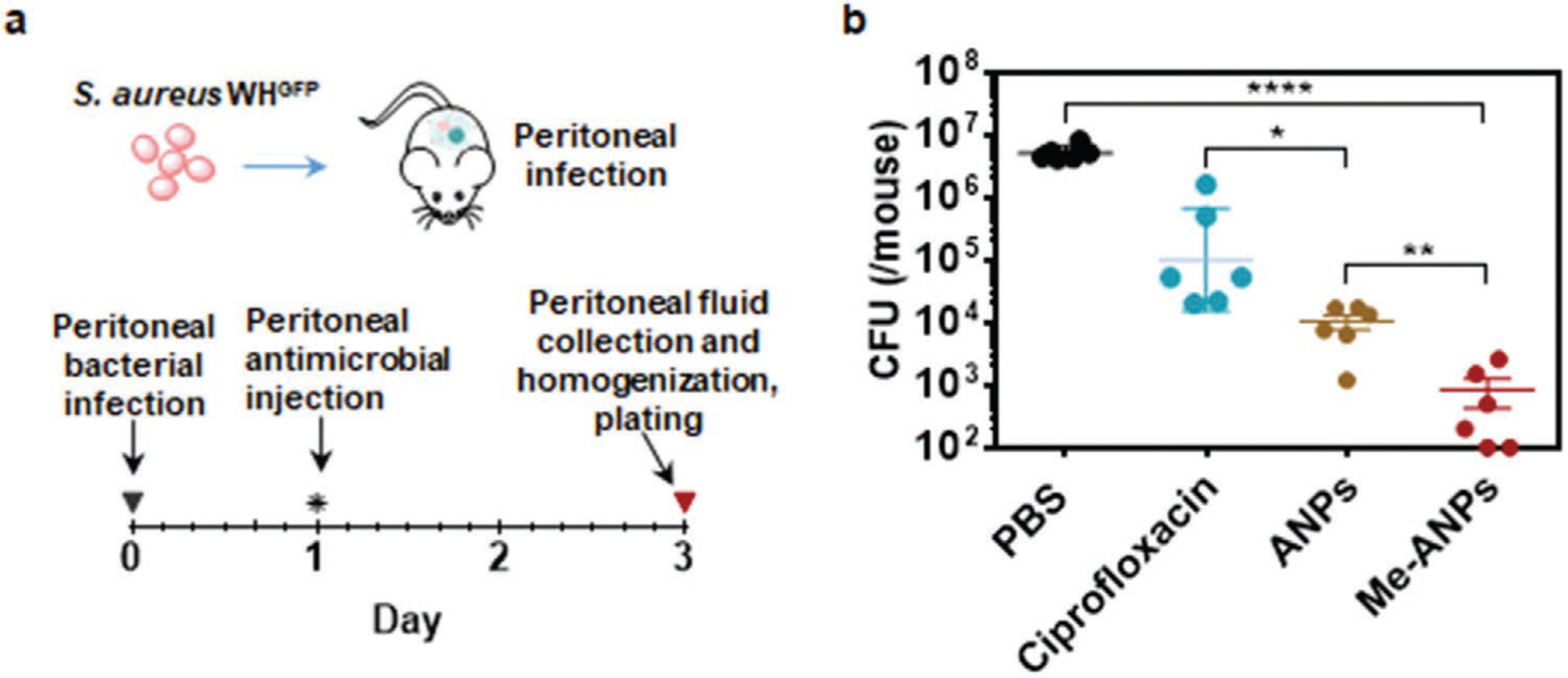
In vivo antimicrobial efficacy of mouse J774A.1 membrane-encapsulated, antimicrobial-conjugated nanoparticles compared with PBS, ciprofloxacin and ANPs with and without membrane-encapsulation, assessed in a mouse, acute peritonitis model. a) Schematics of the mouse, acute peritonitis model used. b) The number of CFUs retrieved from 5 mL peritoneal fluid, extracted 2 d after intraperitoneal antimicrobial injection. Data are presented as means ± standard deviations for 5 mice per group. Asterisks above the data points indicate statistical significance at *p* < 0.05 (*), *p* < 0.01 (**), *p* < 0.001 (***), and *p* < 0.0001 (****), one-way ANOVA test.

Acute peritoneal infection is a life-threatening condition invoking rapid action of macrophages to clear the infection.^[61,62]^ Therefore, we first compared eradication of an acute peritonitis in mice by ciprofloxacin versus ANPs with (Me-ANPs) and without J774A.1 membrane-encapsulation, using PBS as a negative control (Figure 7a). Plating of homogenized intraperitoneal fluid extract (Figure 7b) yielded the highest CFU count after injection of PBS. Injection with ciprofloxacin yielded 2 log-units fewer CFUs than PBS injection, while injection with APNs was a further 1 log-unit more effective in clearing staphylococci from peritoneal fluid than ciprofloxacin. However, treatment by Me-ANPs was most effective in eradicating acute peritonitis, reducing the number of peritoneal CFUs with respect to PBS by 4 log-units, which is significantly more than achieved by ANPs without J774A.1 membrane-encapsulation or by ciprofloxacin.

### 2.8. Eradication of Organ Infection in Mice Using J774A.1-Membrane-Encapsulated, Antimicrobial-Conjugated NPs

**Figure 8.**
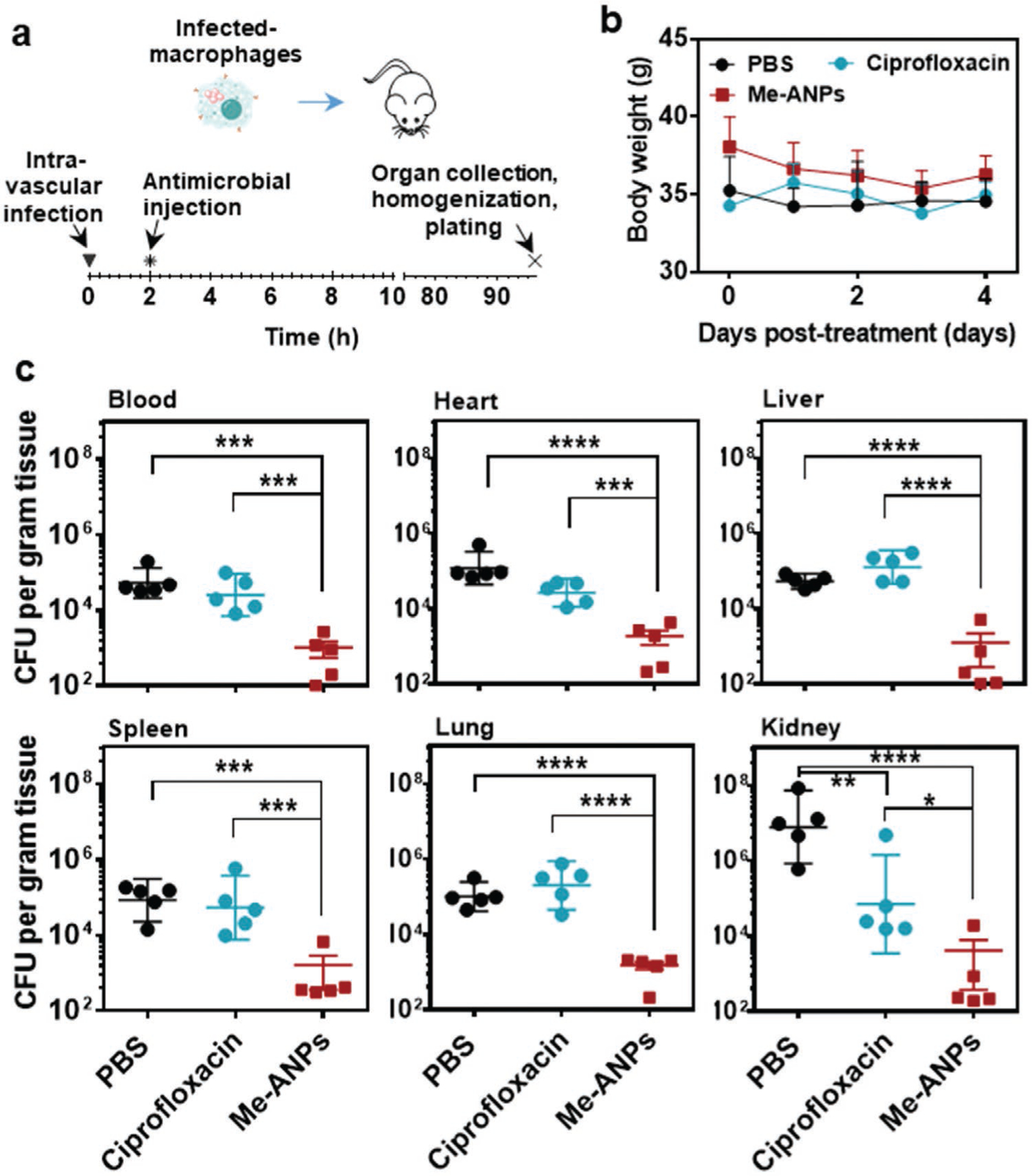
In vivo antimicrobial efficacy of mouse J774A.1 macrophage membrane-encapsulated, antimicrobial-conjugated nanoparticles, assessed in a mouse, intravenous organ infection model. a) Schematics of the mouse, intravenous infection model used. Organ infection was induced by intravenous injection of 200 μL of a suspension of mouse macrophages with intracellular *S. aureus* WH^GFP^, followed after 2 h by intravenous injection of 200 μL PBS, or PBS with ciprofloxacin or Me-ANPs. b) Body weight of the mice as a function of time posttreatment by intravenous injection. Data are presented as means ± standard deviations over 5 mice per group. c) The number of CFUs retrieved from 1 g of homogenized organ tissue for different organs, excised 4 days after intravenous antimicrobial injection. Data are presented as means ± standard deviations over 5 mice per group. Asterisks above the data points indicate statistical significance at *p* < 0.05 (*), *p* < 0.01 (**), *p* < 0.001 (***) and *p* < 0.0001 (****), one-way ANOVA test.

Macrophages with intracellular *S. aureus* can spread via the blood circulation to infect organs and increase the severity of disease.^[16]^ Therefore, we also evaluated the antimicrobial efficacy of mouse J774A.1 Me-ANPs in an established mouse organ infection model^[13]^ after intravenous injection of infected macrophages with in vivo internalized staphylococci to disseminate infection through the body. 2 h after intravenous infection of infected macrophages with intracellular *S. aureus* WH^GFP^, mice were injected with a single dose of PBS, ciprofloxacin, or J774A.1 Me-ANPs. Mice were sacrificed at day four posttreatment after which various organs were removed, homogenized and plated (see Figure 8a). Mice demonstrated no visible adverse effects of Me-ANPs injection, while body weight of the mice showed little variation over the course of the infection period till sacrifice (Figure 8b) in line with the absence of in vitro cytotoxicity observed (Figure S12, Supporting Information). Staphylococcal CFUs retrieved per gram homogenized organ tissue are summarized in Figure 8c for six organs. J774A.1 Me-ANPs demonstrated significant killing by 2–3 log-units relative to PBS per gram organ tissue in all organs. In contrast, ciprofloxacin only showed significant killing of staphylococci disseminated to the kidneys.

## 3. Discussion

Nature-inspired, drug-loaded nanocarriers are emerging as an alternative for synthetic carriers, possessing a biocompatibility that is difficult to achieve with fully synthetic nanocarriers.^[30]^ Here, we developed a novel amphiphilic, binary ANPs encapsulated in macrophage membranes. Binary antimicrobial-conjugates have been made before, like antibody-antibiotic conjugates to target specific pathogens,^[13]^ but the current use of antimicrobial-conjugates that self-assemble through micelle formation into an antimicrobial NP and their stabilization by macrophage membranes is new. Macrophage-monocyte Me-ANPs retained a large variety of membrane proteins and critical Toll-like receptors on their surfaces from their parent cells (Figure 2e and Figure S7e, Supporting Information), including cell membrane TLRs (TLR2 and 4) as well as endosomal membrane TLRs (TLR 3 and 9). Membrane proteins were demonstrated to have been transferred to ANPs in an amount sufficient to exert their activities ^[37,52]^ and functions of transferred proteins were identified to coincide with those of proteins in macrophage-monocyte membranes.

However, apart from maintaining critical membrane components upon isolation and coating, the transverse asymmetry of phospholipids in plasma membrane bilayers (“sidedness”) is functionally important. Particularly the orientation of phos-phatidylserine as an “eat-me signal” or “picking side”, outward projection the N-termini of the TLRs and burial of their C-termini in the lumen are distinctive characteristics of membrane bilayers. Membrane sidedness is actively maintained in parent cells,^[63]^ although the mechanisms by which this is achieved in living cells is not clear. According to Steck and Lange, the fundamental organizing feature of the membrane is the phospholipid bilayer.^[63]^ Polar head groups of phospholipids may be considered as a driving force for organizing membrane sidedness, creating a generally uncharged outer leaflet and strongly negatively charged inner leaflet.^[64,65,66,67,68,69]^ It is likely that phospholipids in membrane fragments constitute a similar driving force in establishing a uniform sidedness in membrane coatings as in cells. This suggestion is confirmed by the relatively little negative zeta potentials of Me-ANPs (−25 mV; see Figure 2h) as compared with zeta potentials of membrane fragments (−47 mV) that represent an average over both membrane sides. Apart from polarity as a driving force for maintaining sidedness in biomimetic membrane coatings, also nanoparticle charge and diameter play a decisive role. Red blood cell membranes covered polymeric nanoparticles with the right side out, but only when the nanoparticles carried a negative charge and possessed a diameter between 65 and 340 nm.^[70]^ The negative zeta potential (Figure 2h) and a diameter within the above range of the ANPs synthesized here (Figure 2b), suggest that our Me-ANPs may also have the right side of the membrane out, despite differences between red blood cell membranes and macrophage membranes.

Absence of cytotoxicity of Me-ANPs was confirmed in vitro based on a metabolic activity assay employing two non-phagocytic cell lines in addition to phagocytic J774A.1 and THP-1 cell lines (Figure S12, Supporting Information). In vivo, when encapsulated with mouse J774A.1 macrophage membranes, there were no obvious adverse effects of Me-ANP after injection in mice. To avoid adverse immunological effects, human THP-1 encapsulated antimicrobial-conjugated NPs were not evaluated in mouse infection models. Engulfment of self-derived compounds occurs during untreated *S. aureus* infection as a result of tissue damage, and this has potential implications for development of autoimmune responses. The novel approach as developed in the current study may differentially impact on how host antigens are processed and presented. Although human THP-1 cells yielded encapsulation of ANPs with similar properties as mouse J774A.1 encapsulated ones, this warrants further investigation, also in view of the rapidly expanding spectrum of bacterial pathogens co-driving autoimmune disease.

**Figure 9.**
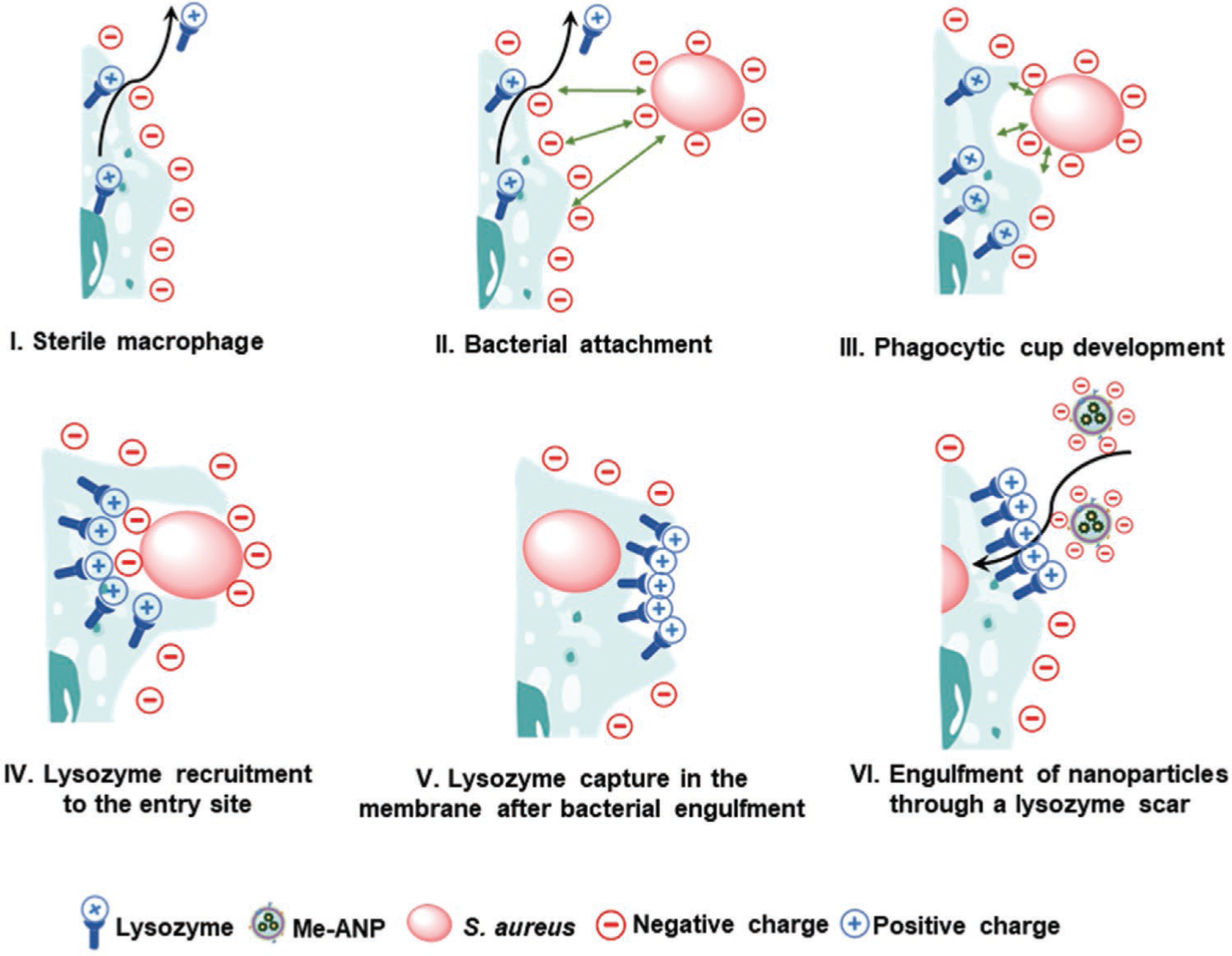
Proposed mechanism of selective engulfment of negatively-charged, macrophage membrane-encapsulated, antimicrobial-conjugated NPs into infected macrophages through lysozyme-rich scars created during staphylococcal engulfment. I) Sterile macrophages excrete lysozyme to kill extra-cellular planktonic bacteria. II) Positively charged lysozyme molecules are recruited to a partly engulfed, negatively charged staphylococcus. III–V) During recruitment, lysozyme is captured in the membrane upon closure of the phagocytic cup to leave a lysozyme-rich, positively charged membrane scar with dimensions comparable with the bacterial diameter, i.e., much larger than the NPs. VI) Positively-charged lysozyme scars attract negatively charged Me-ANPs to the membrane of an infected cell. Sterile cells only possess scattered lysozyme molecules, embedded in a highly negative-charged membrane repelling negatively charged Me-ANPs.

As an unexpected outcome, J774A.1 Me-ANPs were only engulfed by infected macrophages and not by sterile cells without internalized staphylococci. Enhanced engulfment of modified, antimicrobial-loaded liposomes has been reported,^[15,23,24]^ but the authors point out that the engulfment process itself remained poorly understood,^[24]^ leaving many questions open. Providing a mechanism for selective engulfment observed for macrophage Me-ANPs is accordingly even more difficult, but based on the differences measured for sterile and infected J774A.1 macrophages relevant for interaction with membrane-encapsulated, antimicrobial-conjugated nanoparticles, we provide a model supported by a variety of data collected and shown in Figure 2 (zeta potentials and abundance of Toll-like receptors), Figure 5 and Figure S11, Supporting Information (lysozyme presence on cells after bacterial engulfment) that is schematically summarized in Figure 9.

Staphylococcal attachment to J774A.1 cells occurs through attractive Lifshitz-Van der Waals and specific ligand-receptor forces, despite electrostatic double-layer repulsion between the negatively-charged staphylococci and the J774A.1 cell surface (Figure 9, panel II). Thus, whereas sterile macrophages excrete lysozyme (Figure 9, panel I), data suggest that positively-charged intracellular lysozyme molecules are recruited during engulfment (Figure 9, panel III) of a negatively-charged staphylococcus and subsequent captured in the membrane upon closure of the phagocytic cup,^[57]^ (Figure 9, panel IV). This process leaves a positively-charged, lysozyme-rich membrane scar (Figure 9, panel V). The dimensions of these scars are expected to be comparable or slightly smaller than the bacterial diameter of around 1 μm, but ≈10-fold larger than the Me-ANPs. The localized nature of these lysozyme-rich membrane scars follows from the overall negative zeta potential of the membrane from infected cells: positively charged lysozyme-rich scars will attract small, negatively charged Me-ANPs through electrostatic double-layer attraction (Figure S9, Supporting Information),^[71]^ yielding their engulfment (Figure 9, panel VI). Sterile cells only possess scattered lysozyme molecules (impossible to visualize using fluorescently labeled antibodies), embedded in a highly negative-charged membrane (Figure 9, panel I), thus overall repelling negatively charged Me-ANPs. Therewith membrane approach and engulfment of negatively charged Me-ANPs toward sterile cells is inhibited by electrostatic-double layer repulsion. Selective internalization avoids unnecessary consumption of Me-ANPs, and presents a potential advantage for clinical application, as healthy leukocytes in which no staphylococci are hiding, will not be compromised. Experiments with macrophages and monocytes that are defective for lysozyme production, for instance as derived from lysM knockout mice, might confirm the mechanism proposed in Figure 9. Furthermore, it will be valuable to assess whether the selective internalization mechanism of Me-ANPs established here for macrophages-monocytes is also operational for low-phagocytic cells, types such as endothelium and other epithelia where *S. aureus* or other pathogens take up residence, sequestered away from immune cells. Other mechanistic work downstream of the novel lysozyme cupping mechanism we identify here, includes elucidation how and in which intracellular compartments the antimicrobials are actually liberated. Intracellular compartments are unlikely to mingle, while Lacoma et al.^[12]^ have shown that *S. aureus* is present in acidic subcellular compartments expressing markers for late endosomes/lysosomes, like LAMP-1, Rab7 and RILP.

After engulfment by infected macrophages, the antimicrobial-conjugates kill infecting staphylococci more effectively than ciprofloxacin. In fact, in vivo ciprofloxacin yielded similarly low staphylococcal killing in an organ infection model (Figure 8c) as did PBS as a negative control. This is probably because ciprofloxacin, though able to enter mammalian cells and sub-compartments such as phagosomes, is inactivated in the acidic environment of phagosomes.^[72]^ This inactivation mechanism makes it likely that in our Me-ANPs, triclosan does the actual killing of pathogens in infected leukocytes, as triclosan is highly stable compared with ciprofloxacin. Its anaerobic degradation occurs not within 70 d, while aerobic degradation requires at least 18 d.^[73]^ Importantly, the use of triclosan in soaps, detergents, clothing, carpets, toys, school supplies and pacifiers is under heavy debate, but its use in healthcare is undisputed.^[74]^

## 4. Conclusion

In summary and perspective, we have demonstrated for the first time that binary, amphiphilic antimicrobial-conjugates self-assemble into antimicrobial NPs that can be stabilized for several weeks at pH 7.4 by encapsulation in mouse J774A.1 or human THP-1 membranes, as models for macrophages and monocytes, respectively. Acute peritonitis induced by staphylococcal injection was more effectively eradicated by mouse macrophage membrane-encapsulated ANPs than by ANPs in absence of membrane-encapsulation, while both performed better than ciprofloxacin, the current clinical standard. In addition, mouse macrophage Me-ANPs showed up to 4 log-units higher killing of staphylococci in various organs of mice, infected through hematogenous dissemination of macrophages with internalized staphylococci, than ciprofloxacin. Moreover, no adverse effects were observed upon intravenous injection of Me-ANPs and their circulation in the blood stream. Along similar lines, very recently, peritoneal liposomal vancomycin was demonstrated to reduce *S. aureus* kidney infection and mouse mortality, even when administered a day after infection.^[11]^ Innovative lysosomal carriers are also being developed for tuberculosis therapy,^[75]^ including liposomes for antibiotic delivery and immunization.^[76]^ In addition, nanoparticles can be employed for tuberculosis therapy by promoting phagolysosome fusion (by metformin), phagosome acidification and maturation (imatinib), by inhibition of phagolysosome fusion (ManLAM), and by phagosome destabilization (ESX-1).^[77]^

For future large-scale clinical application, an off-the-shelf product can be envisioned based on a human monocyte cell line, preferably with very low or absent expression of major human leukocyte antigens to prevent alloimmunization.^[78]^ Individualized treatment can be based on peripheral blood monocytes, but costs would be proportionally higher. Thus, leukocyte Me-ANPs can become a powerful means to eradicate intracellular bacteria hiding in macrophages, for which there is currently no effective control strategy available in daily clinical practice.

## 5. Experimental Section

### Synthesis of an Amphiphilic, Binary Antimicrobial Conjugate:

A mixture of triclosan (2.89 g, 10 mmol, 1.0 equivalent), triethylamine (Et_3_N, 1.11 g, 11 mmol, 1.1 equivalent) in anhydrous dichloromethane (CH_2_Cl_2_, 100 mL) was cooled to −5 °C. Chloroacetyl chloride (1.13 g, 10 mmol, 1.0 equivalent) in dry dichloromethane (20 mL) was added drop wise to this reaction mixture under stirring over a period of 1 h at −5 °C. Next, the reaction mixture was stirred overnight at room temperature, diluted with dichloromethane (100 mL), washed with 5% HCl (100 mL, 1×) and 5% sodium hydroxide solution (100 mL, 1×).

The organic layer was washed with saturated aqueous NaCl, dried over anhydrous magnesium sulfate, filtered and solvent was removed under reduced pressure. The crude product was purified on a silica gel column to yield the triclosan chloroacetyl derivative as a colorless oil (yield: 82%). ^1^H NMR (400 MHz, CDCl_3_) *δ* 7.55 (s, 1H), 7.37−7.23 (m, 3H), 6.99 (d, *J* = 7.0 Hz, 1H), 6.89 (d, *J* = 7.0 Hz, 1H), 4.34 (s, 2H). ^13^C NMR (100 MHz, CDCl_3_) *δ* 165.02, 150.59, 146.73, 141.01, 130.61, 130.14, 129.22, 128.38, 127.65, 126.40, 124.05, 120.99, 119.79, 40.35 (Figure S1, Supporting Information).

**Figure 10.**
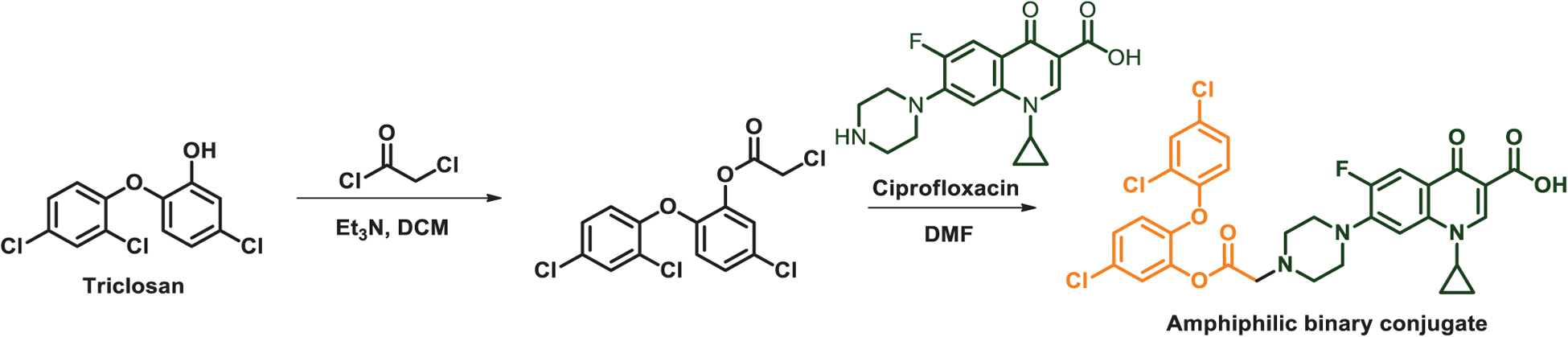
Conjugation of hydrophobic triclosan and hydrophilic ciprofloxacin.

The triclosan chloroacetyl derivative (336 mg, 1.0 mmol), trimethylamine (0.416 mL, 3.0 mmol) and potassium iodide (249 mg, 1.5 mmol) were added to a solution of ciprofloxacin (331 mg, 1.0 mmol) in dimethylformamide (5 mL) under argon atmosphere. The mixture was stirred at room temperature overnight and subsequently poured into water (50 mL). The resulting precipitate was filtered off, washed with water and recrystallized from methanol. Ciprofloxacin–triclosan conjugate (see Figure 10) was obtained as a yellow powder (560 mg, 85%). ^1^H NMR (400 MHz, DMSO) *δ* 15.20 (s, 1H), 8.66 (s, 1H), 7.89 (d, *J* = 13.1 Hz, 1H), 7.76 (d, *J* = 2.5 Hz, 1H), 7.55 (t, *J* = 7.1 Hz, 2H), 7.40 (dd, *J* = 8.8, 2.5 Hz, 2H), 7.16 (d, *J* = 8.8 Hz, 1H), 6.96 (d, *J* = 8.9 Hz, 1H), 3.81 (s, 1H), 3.55 (s, 2H), 2.73 (s, 4H), 1.31 (d, *J* = 6.7 Hz, 2H), 1.17 (s, 2H), ^13^C NMR (100 MHz, DMSO) *δ* 176.80, 168.18, 166.38, 151.23, 148.38, 146.51, 145.56, 141.87, 139.59, 130.56, 129.26, 128.94, 128.87, 127.97, 125.11, 124.93, 121.92, 120.67, 111.51, 111.28, 106.81, 58.15, 51.78, 49.82, 49.77, 36.29, 8.05. MALDI-TOF MS (m/Z, [M+H]^+^, calculated: 660.0866, 662.0836, 661.0889, 663.0870, 664.0807; found: 660.0869, 662.0841, 661.0933, 663.0888, 664.0795) (Figure S2, Supporting Information).

The critical micelle concentration (CMC) of the antimicrobial conjugates was measured using pyrene as a fluorescence probe. To this end, pyrene (0.6 × 10^−3^ m) was dissolved in acetone and 10 μL aliquots were added into tubes allowing evaporation of acetone and mixed with conjugate solution (450 μL) with different concentrations under overnight shaking in dark. Fluorescence emission spectra between 350 and 500 nm were recorded on a fluorescence spectrometer (Hitachi F-4600, Tokyo, Japan) at an excitation wavelength of 334 nm. The change in linearity between the fluorescence intensity ratios at 373 nm and 384 nm (*I*_373_/*I*_384_) and antimicrobial conjugate concentration was taken as the conjugate CMC.^[79]^

### Isolation of Mouse J774A.1 and Human THP-1 Cell Membranes:

Mouse J774A.1 (ATCC TIB-67) and human THP-1 (ATCC TIB-202) cells were purchased from American Type Culture Collection to model mouse macrophages and human monocytes, respectively. Leukocytes were cultured in Dulbecco’s modified Eagle’s medium (high glucose, Gibco, Thermo Fisher Scientific, MA, USA) supplemented with 10% fetal bovine serum (FBS, Gibco) and 0.1% L-ascorbic acid 2-phosphate sesquimagnesium salt (AA2P, Sigma) at 37 °C in a humidified 5% CO_2_ incubator.

In order to isolate cell membranes, cells were harvested using a cell scraper at 80–90% confluency and resuspended at a concentration of 2.5 × 10^7^ cell mL^−1^ in ice-cold tris-magnesium buffer (TM buffer, pH 7.4, 0.01 m tris and 0.001 m MgCl_2_) and 1 EDTA-free mini protease inhibitor tablet (Pierce, ThermoFisher Scientific) per 10 mL of suspension. Cells were denucleated using a sonicator (Vibra cell model 375, Sonics and Material, Inc., Danbury, CT) for 4 × 10 s, while cooling in an ice/water bath. The homogenate was mixed with 1 m sucrose to a final concentration of 0.25 m sucrose, and centrifuged at 2000 *g* and 4 °C for 10 min. The supernatant was collected and further centrifuged at 20 000 *g* and 4 °C for 30 min in order to collect leukocyte membranes. Membranes were washed with ice-cold TM-buffer with 0.25 m of sucrose and collected by centrifugation at 20 000 *g* and 4 °C for 30 min and stored at −20 °C for further use.

### Antimicrobial-Conjugated Nanoparticles (ANPs) Preparation and Macrophage-Monocyte Membrane-Encapsulation:

For the preparation of ANPs, 200 μL of binary antimicrobial-conjugate stock solution in dimethylsulfoxide (10 mg mL^−1^) was added dropwise to 5 mL ultrapure water under magnetic stirring (2500 rpm) to form an ANP suspension through self-assembly, occurring by virtue of their amphiphilic character. After further stirring for 30 min, the ANPs suspension was centrifuged at 20,000 *g* (centrifuge 5417R, Eppendorf, Germany) and 4 °C for 30 min to collect the ANPs. Then, ANPs were rinsed once with ultrapure water (5 mL) to remove the residual organic solvent. Finally, the ANPs were resuspended in 2 mL ultrapure water to a final concentration of 1 mg mL^−1^ and stored at 4 °C for further use.

For macrophage-monocyte membrane-encapsulation, ANP suspensions (2 mL) were mixed with membranes isolated from a total of 5 × 10^6^ leukocytes. The resulting mixture was homogenized using a sonicator for 4 × 10 s while cooling in an ice/water bath. The encapsulated ANPs were centrifuged at 20 000 g and 4 °C for 30 min and washed once with cold ultrapure water. Finally, membrane-encapsulated (Me) ANPs were collected and resuspended in 2 mL ultrapure water to a final concentration of 1 mg mL^−1^ and stored at 4 °C for further use.

### Nanoparticle Characterization, Stability and Antimicrobial-Conjugate Release:

Transmission electron microscopy (TEM) was performed to demonstrate the presence of membrane-encapsulation and ANPs stability, using a Glacios Cryo-TEM (Thermo Scientific, Massachusetts, United States) at an acceleration voltage of 200 kV. TEM samples were prepared by applying a droplet of a ANPs or Me-ANPs suspension onto a carbon coated copper grid and drying it at room temperature.

Hydrodynamic diameters of the ANPs with or without membrane-encapsulation were measured at 25 °C using a Zetasizer Nano-ZS (Malvern Instruments, Worcestershire, UK) in 10 × 10^−3^ m potassium phosphate buffer as a function of storage time in buffer. Similarly, zeta potentials and polydispersity indices (PDI) of the ANPs were measured using the same instrument. Both types of measurements were done at an ANP concentration of 0.1 mg mL^−1^. Drug-loading in ANPs was measured using UV–vis absorption spectroscopy (Shimadzu, Japan). First absorption spectra were taken at different antimicrobial-conjugate concentrations (Figure S6a, Supporting Information) and a calibration curve (Figure S6b, Supporting Information) was prepared using the unique absorption peak of the antimicrobial-conjugate at 281 nm for quantitation (Figure S6c, Supporting Information). The UV–vis calibration curve was also applied to determine the release of conjugates from the ANPs in absence and presence of membrane-encapsulation. To this end, 2 mL ANP suspension (1.0 mg mL^−1^) was transferred into a dialysis bag (molecular weight cut off: 12–14 kDa) and subsequently immersed in 20 mL PBS (pH 7.4 and pH 5.0) at 37 °C. Aliquots (1 mL) of the dialysis solution were collected every 30 min up to 72 h, and the absorbance of the solutions at 281 nm was recorded. The volume of the stock dialysis solution was kept constant by adding 1 mL of fresh buffer, after each aliquot was taken.

In order to identify the proteins on the macrophage-monocyte membranes isolated and on Me-ANPs, proteomic analyses were employed. Proteins were precipitated from membranes or Me-ANPs using the ProteoExtract Kit (Merck, Darmstadt, Germany), as described in the user guide. Precipitated proteins were solubilized in 25 × 10^−3^ m ammonium bicarbonate containing 0.1% RapiGest (Waters, Eschborn, Germany) at 80 °C for 15 min. Proteins were reduced by adding 5 × 10^−3^ m DTT (45 min, 56 °C), and free cysteines alkylated with iodoacetamide (Sigma, Taufkirchen, Germany) (15 × 10^−3^ m, 25 °C, 1 h in the dark). 0.2 μg porcine sequencing grade trypsin (Promega, Mannheim, Germany) was added and samples incubated overnight at 37 °C. After incubation, RapiGest was hydrolyzed by adding 10 × 10^−3^ m HCl (37 °C, 10 min). The resulting precipitate was removed by centrifugation (13 000 g, 15 min, 4 °C), and the supernatant was transferred into an autosampler vial for peptide analysis using liquid chromatogrpahy-mass spectroscopy (LC-MS).^[52]^ LC-MS data were employed in UniProt/GO^[51]^ to determine characteristic functions of the membrane proteins.

### Staphylococcal Culturing and Harvesting:

Two multidrug-resistant staphylococcal strains^[45]^ were employed in this study: green-fluorescent *S. aureus* WH^GFP^ and commercially available bioluminescent *S. aureus* Xen36 (PerkinElmer Inc., Waltham, MA, USA). Both strains were cultured at 37 °C in ambient air. *S. aureus* WH^GFP^ was cultured on Tryptone soy broth (TSB, OXOID, Basingstoke, UK) agar plates with 10 μg m^−1^ tetracycline, while *S. aureus* Xen36 was cultured on agar plates with 200 μg mL^−1^ kanamycin. One colony was inoculated in 10 mL TSB medium with 10 μg mL^−1^ tetracycline for *S. aureus* WH^GFP^ and for *S. aureus* Xen36 TSB and incubated for 24 h at 37 °C, this preculture was inoculated (1:20) into 200 mL main culture and grown for 16 h. Bacterial cultures were harvested by centrifugation for 5 min at 5000*g*, washed twice in PBS and sonicated for three times 10 s (Vibra cell model 375, Sonics and Material Inc., Danbury, CT), while cooling in an ice/water bath to break possible aggregates. Finally, suspensions were diluted in PBS to concentrations required in the respective experiments, as determined in a Bürker-Türk counting chamber.

### Engulfment of Macrophage-Monocyte Membrane-Encapsulated, Antimicrobial-Conjugated Nanoparticles into J774A.1 Macrophages In Vitro: For Engulfment Studies:

Nile red-loaded, Me-ANPs were prepared, essentially as described above for the preparation of ANPs. However, during ANP preparation, Nile red in dimethylformamide (40 μL, 1 mg mL^−1^) was added. For comparison, Nile red-loaded PC-liposomes were included, prepared by mixing a Nile red stock solution in dimethylformamide (40 μL, 1 mg mL^−1^) with phosphocholine-based phospholipids (DPPC, DSPC and DOPC) and cholesterol (Avanti Polar Lipids) in a chloroform:methanol mixture (200 μL, 3:1 v/v, 5 mg mL^−1^) to a total volume of 2 mL in PBS.

To study engulfment of macrophage-monocyte Me-ANPs and PC-liposomes in sterile and bacterially infected J774A.1 macrophages, infected cells were prepared by in vitro internalization of staphylococci. Briefly, J774A.1 cells were seeded at a density of 4 × 10^5^ cells mL^−1^ in six-well-plates and grown for 12 h at 37 °C in a 5% CO_2_ incubator. Subsequently, the cell-culture was infected with *S. aureus* WH^GFP^ at a ratio of 20 bacteria per cell and supplemented with 100 μg mL^−1^ of gentamicin, as a widely used tool to prevent growth of extracellular staphylococci. After coculturing for 24 h at 37 °C in a 5% CO_2_ incubator, the wells were gently emptied and washed once with 1 mL PBS to remove non-internalized staphylococci. After removal of growth medium, 2 mL freshly prepared Nile red-loaded Me-ANPs or PC-liposomes (0.1 mg mL^−1^ in PBS, see above) were added to each well, followed by culturing at 37 °C for 2 h. Subsequently, the cell culture was rinsed by PBS (1 mL per well), fixed with 3.7% paraformaldehyde for 15 min at room temperature and permeabilized with 0.1% Triton X-100 (1 mL well^−1^) in PBS. Nuclei were stained with 4’,6-diamidino-2-phenylindole (DAPI) in PBS (4 μg mL^−1^) for 1 h at room temperature. J774A.1 cells with internalized red-fluorescent Me-ANPs or PC-liposomes were observed using a CLSM.

For CLSM, a TCS SP2 (Leica, Wetzlar, Germany) was used, equipped with an argon ion laser at 488 nm to excite green-fluorescent staphylococcal protein and a 543 nm green neon laser to excite Nile red. A solid-state 405 nm laser was used for excitation of DAPI. Fluorescence signals were detected at 430–500 nm (blue, cellular nuclei), 500–535 nm (green, staphylococci) and 583–688 nm (red, due to Nile red in Me-ANPs). All data were acquired and analyzed using Leica software, version 2.0 and Image J software.

### Lysozyme Distribution on Sterile and Infected Macrophage-Monocyte Membranes:

Lysozyme distribution on sterile and infected macrophages-monocytes was compared using immunofluorescence staining immediately and 12 h after infection (only for J774A.1 cells). For immediate analysis using CLSM, sterile cells and cells infected with green-fluorescent *S. aureus* WH^GFP^ (see above), cells were fixed in 4% paraformaldehyde for 15 min at room temperature, washed three times with PBS and exposed to 5% fetal bovine serum for 1 h, followed by staining with lysozyme primary antibodies (rabbit anti-lysozyme antibody, Abcam, Cat#EPR2994(2)) 16 h at 4 °C, according to the manufacturer’s instructions. Anti-lysozyme antibodies were applied for labeling in absence of Triton X-100 in order to avoid entry of anti-lysozyme antibodies into the cell and labeling of lysozyme inside the cell.^[59]^ Subsequently, fluorescently labeled secondary antibodies (Donkey anti-Rabbit, Invitrogen, Cat#A32754) were added for 1.5 h. All antibodies were applied at concentration 5 μg mL^−1^ in PBS. Finally, the nuclei of macrophages were stained with blue-fluorescent DAPI. Lysozyme distribution was observed using CLSM, as described above. For analysis of the time-dependence of lysozyme presence, infected macrophages were analyzed by flow cytometry. Sterile cells and cells infected with green-fluorescent *S. aureus* WH^GFP^, were washed three times with PBS and followed by staining with lysozyme primary antibodies 0.5 h at room temperature, according to the manufacturer’s instructions and washed three times. Subsequently, fluorescently labeled secondary antibodies were added for 0.5 h at room temperature, after washing three times, FACS was used to quantify red-fluorescent lysozyme presence.

### Antimicrobial Efficacy of Macrophage-Monocyte Membrane-Encapsulated, Antimicrobial-Conjugated Nanoparticles In Vitro:

To determine the MIC of macrophage-monocyte Me-ANPs and their composing antimicrobials, 100 μL of each in PBS (triclosan, ciprofloxacin, 1:1 mixture of triclosan and ciprofloxacin, Me-ANPs, all at equivalent antimicrobial concentrations between 0 and 80 μg mL^−1^) were applied to 100 μL suspension of *S. aureus* WH^GFP^ or *S. aureus* Xen36 in PBS (2 × 10^6^ bacteria mL^−1^). MIC values were taken as the lowest concentration at which visible bacterial growth was absent. Subsequently, the MBC values were determined by plating aliquots of suspensions with concentrations yielding no visible growth of bacteria on TSB agar plates. The lowest concentration at which no colony forming units appeared after 24 h growth at 37 °C was taken as the MBC.

### In Vitro Killing of Staphylococci Hiding in Macrophages by Mouse J774A.1 Membrane-Encapsulated, Antimicrobial-Conjugated Nanoparticles:

In order to compare the intracellular killing efficacy by mouse J774A.1 Me-ANPs with ciprofloxacin and PBS as a control, infected J774A.1 macrophages were exposed for 24 h to Me-ANPs, ciprofloxacin or PBS, similarly as described under “Engulfment of membrane-encapsulated, antimicrobial nanoparticles into leukocytes in vitro” (see above). After 24 h, cells were lysed in PBS supplemented with 0.1% bovine serum albumin and 0.1% Triton-X. Lysate was homogenized by sonication on a JY98-IIIDN (Scientz, Ningbo, China), and serial dilutions of the lysate were made in PBS containing 0.05% Tween-20. The number of staphylococci surviving inside macrophages was determined by plating on tryptic soy agar with 5% defibrinated sheep blood.

### Mouse Acute Peritonitis Model for Evaluating the Killing of Staphylococci by Mouse J774A.1 Membrane-Encapsulated, Antimicrobial-Conjugated Nanoparticles:

Eight week old female mice, ICR (CD-1) (35 g to 40 g each) were obtained from Vital River Laboratory Animal Technology Co. (Beijing, China). All animals were housed in the on-site animal facility of Nankai University and experimental procedures were approved by the Institutional Animal Care and Use Committee of Nankai University, Tianjin, China. Each mouse was injected with a dose of 2 × 10^8^ *S. aureus* WH^GFP^ directly into the peritoneal cavity. At day 1 after bacterial injection, infected animals were randomly assigned into four groups of five animals each, receiving intraperitoneal injection of i) 200 μL PBS (negative control), ii) 200 μL ciprofloxacin in PBS (1 mg mL^−1^), iii) 200 μL ANPs without membrane-encapsulation in PBS (1 mg mL^−1^) and iv) 200 μL Me-ANPs with membrane-encapsulation in PBS (1 mg mL^−1^). Treatment was initiated 1 day post-injection and continued for 2 consecutive days. All mice were killed on day 3 after injection. Peritoneal macrophages were harvested by washing the peritoneal cavity with 5 mL cold PBS. Macrophages were lysed by homogenization on a JY98-IIIDN sonicator (Scientz, Ningbo, China), and serial dilutions of the lysate were made in PBS containing 0.05% Tween-20. The number of surviving peritoneal bacteria was determined by plating on tryptic soy agar plates supplemented with 5% defibrinated sheep blood.

### Mouse Organ Infection Model for Evaluating the Killing of Staphylococci by Mouse J774A.1 Membrane-Encapsulated, Antimicrobial-Conjugated Nanoparticles:

Peritoneal macrophages with infecting staphylococci from different pairs of donor mice were pooled, and injected intravenously into the tail vein of receptor mice in order to allow hematogenous spreading and dissemination of staphylococci to cause organ infection. Infected mice were randomly assigned into three groups of five animals, each receiving intravenous injection of i) 200 μL PBS, ii) 200 μL ciprofloxacin in PBS (1 mg mL^−1^), iii) 200 μL mouse J774A.1 Me-ANPs suspended in PBS (1 mg mL^−1^). All animals were sacrificed on day 4 after infection. Blood, heart, liver, spleen, lungs, and kidneys were harvested in 5 mL of sterilized PBS. Organs were homogenized using a JY98-IIIDN sonicator and the number of surviving bacteria per gram homogenized organ tissue was determined by plating serial dilutions of the tissue homogenates in PBS with 0.05% Tween on tryptic soy agar with 5% defibrinated sheep blood.

## Supplementary Material

ESI
