# Supplementary material for: Coating of a Novel Antimicrobial Nanoparticle with a Macrophage Membrane for the Selective Entry into Infected Macrophages and Killing of Intracellular Staphylococci
Source: Adv Funct Mater. Author manuscript; Available in PMC 2021 Nov 25. (PMC8562776; doi:10.1002/adfm.202004942)
Supplement: ESI [file NIHMS1750863-supplement-ESI.pdf]

Supporting Information

Coating of a Novel Antimicrobial Nanoparticle with a Macrophage Membrane Coating for the Selective Entry into Infected Macrophages and Killing of Intracellular Staphylococci

Yuanfeng Li,[1,2#] Yong Liu,[1,2#] Yijin Ren,[3] Linzhu Su,[1,2] Ang Li,[1] Yingli An,[1] Vincent Rotello,[4] Zhanzhan Zhang[1], Yin Wang[1], Yang Liu[1], Sidi Liu[5], Jian Liu[5], Jon D. Laman[6], Linqi Shi,[1]* Henny C. van der Mei,[2]* Henk J. Busscher[2]

Y. Li, Y. Liu, L. Su, Y. An, Dr. A. Li, Z. Zhang, Y. Wang, Prof. Y. Liu, Prof. L. Shi
[1] State Key Laboratory of Medicinal Chemical Biology, Key Laboratory of Functional Polymer Materials, Ministry of Education, Institute of Polymer Chemistry, College of Chemistry, Nankai University, 94 Weijin Road, Tianjin 300071, P. R. China
E-mail: shilinqi@nankai.edu.cn

Y. Li, Y. Liu, L. Su, Prof. H.C. van der Mei, Prof. H.J. Busscher
[2] University of Groningen and University Medical Center Groningen, Department of Biomedical Engineering, Antonius Deusinglaan 1, 9713 AV Groningen, The Netherlands.
E-mail: h.c.van.der.mei@umcg.nl

Prof. Y. Ren
[3] University of Groningen and University Medical Center Groningen, Department of Orthodontics, Hanzeplein 1, 9700 RB Groningen, The Netherlands

Prof. V. Rotello
[4] Department of Chemistry, University of Massachusetts, 710 North Pleasant Street, Amherst, Massachusetts 01003, United States

S. Liu, Prof. J. Liu
[5] Institute of Functional Nano & Soft Materials (FUNSOM), Jiangsu Key Laboratory for Carbon-Based Functional Materials & Devices, Collaborative Innovation Center of Suzhou Nano Science and Technology, Soochow University, 199 Ren'ai Rd, Suzhou 215123, Jiangsu, P. R. China

Prof. J.D. Laman
[6] University of Groningen and University Medical Center Groningen, Department of Biomedical Sciences of Cells and Systems, Antonius Deusinglaan 1, 9713 AV Groningen, The Netherlands

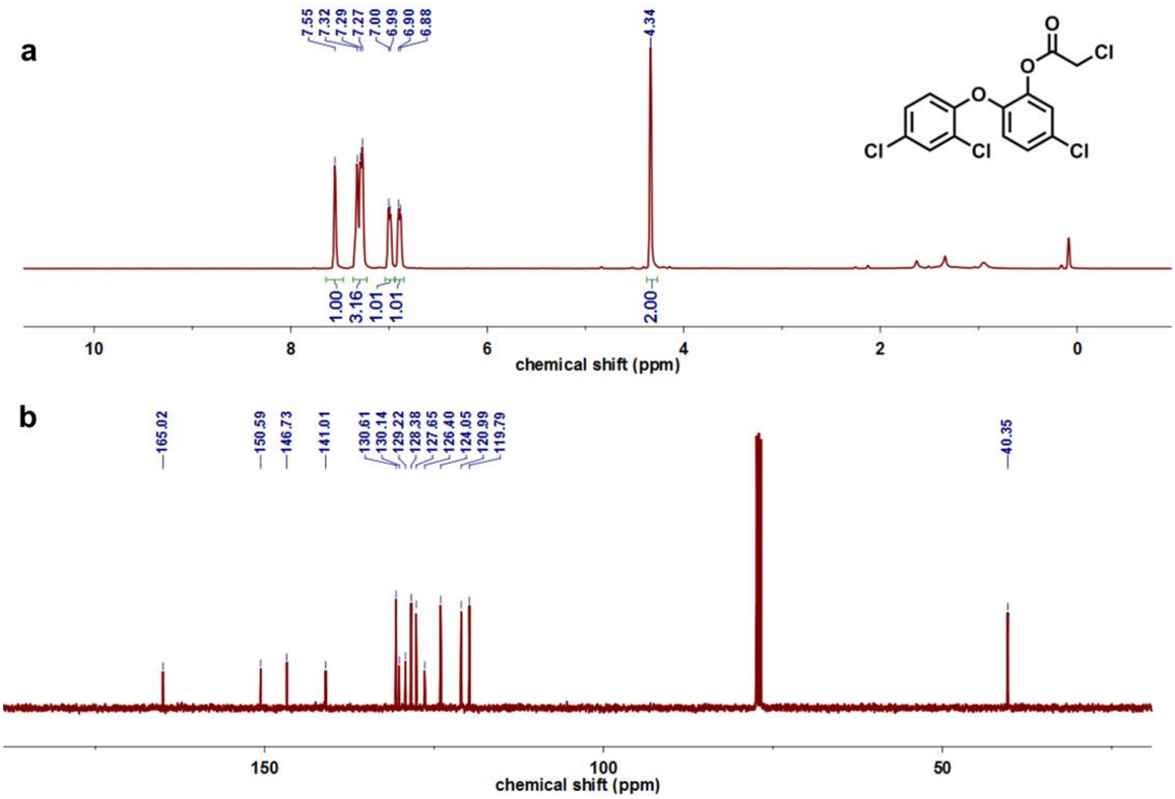

Figure S1. NMR spectra of chloroacetylated Triclosan in *d*-$CHCl_3$ at 0°C. (a) ^{1}H NMR. (b) ^{13}C NMR.

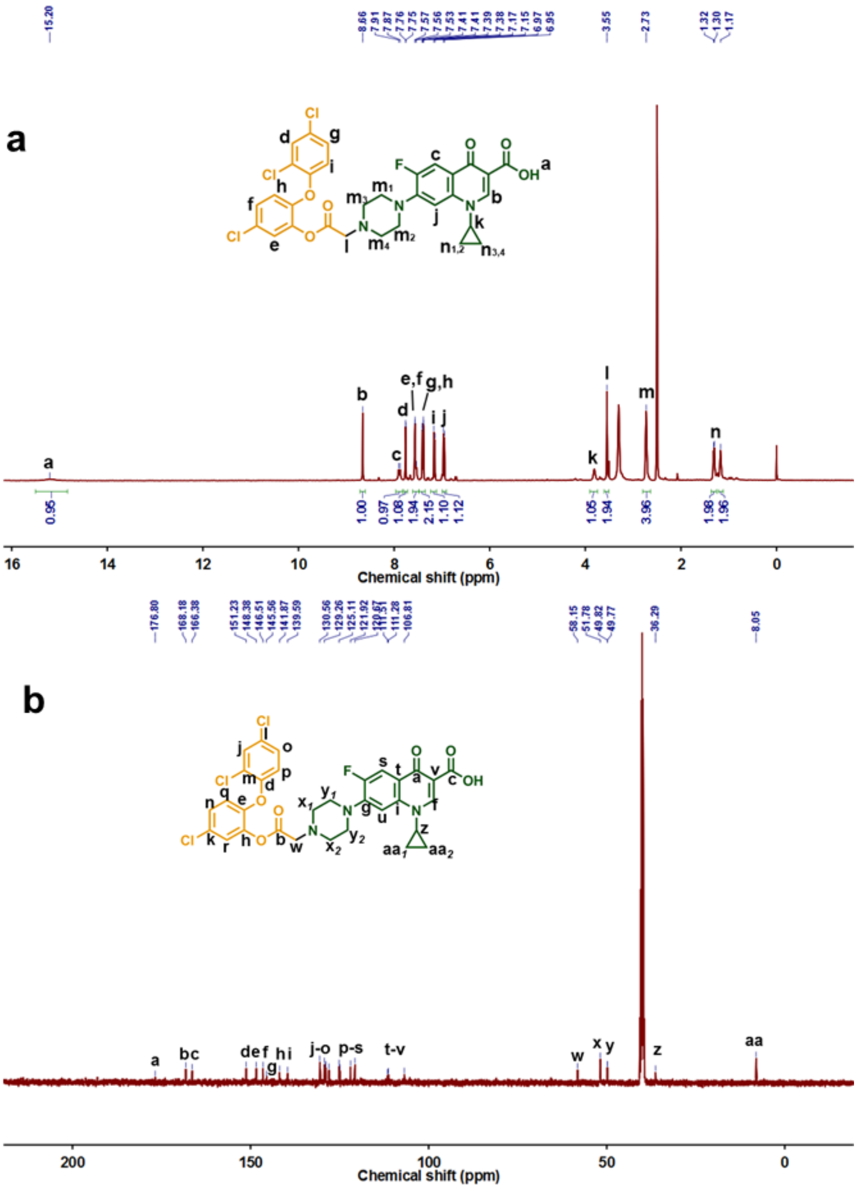

Figure S2. NMR spectra of the amphiphilic, binary antimicrobial-conjugate synthesized, composed of the hydrophobic antimicrobial Triclosan and the hydrophilic antibiotic ciprofloxacin in d_6-DMSO at 0°C. (a) ^{1}H NMR. (b) ^{13}C NMR.

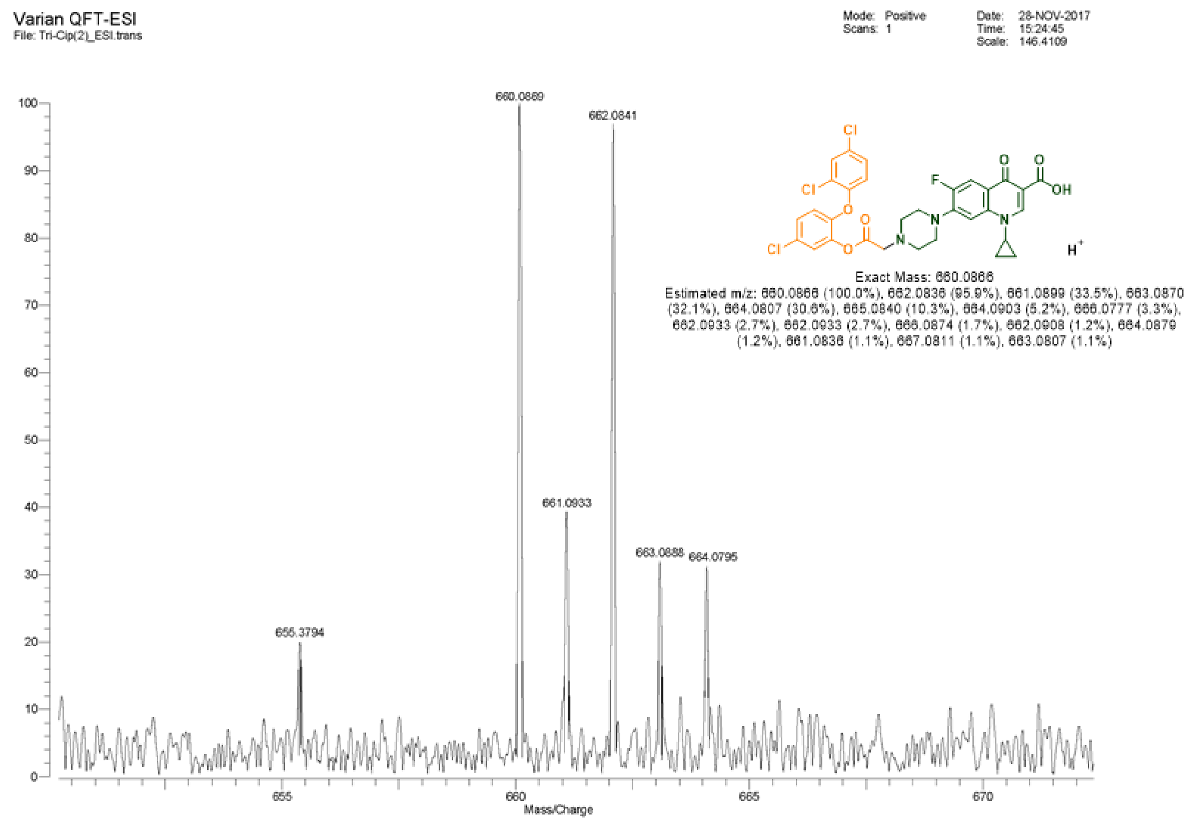

Figure S3. Electrospray ionization (ESI) mass spectrum of the amphiphilic, binary antimicrobial-conjugate synthesized, measured in DMSO.

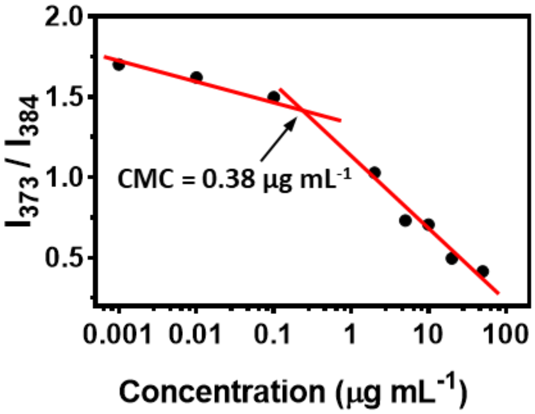

Figure S4. Fluorescence intensity ratio I_{373}/I_{384} of the antimicrobial conjugates prepared as a function of the conjugate concentration in a pyrene/acetone mixture. The critical micelle concentration (CMC) was taken as the conjugate concentration where the two linear sections of the curve intersect (indicated by the arrow).

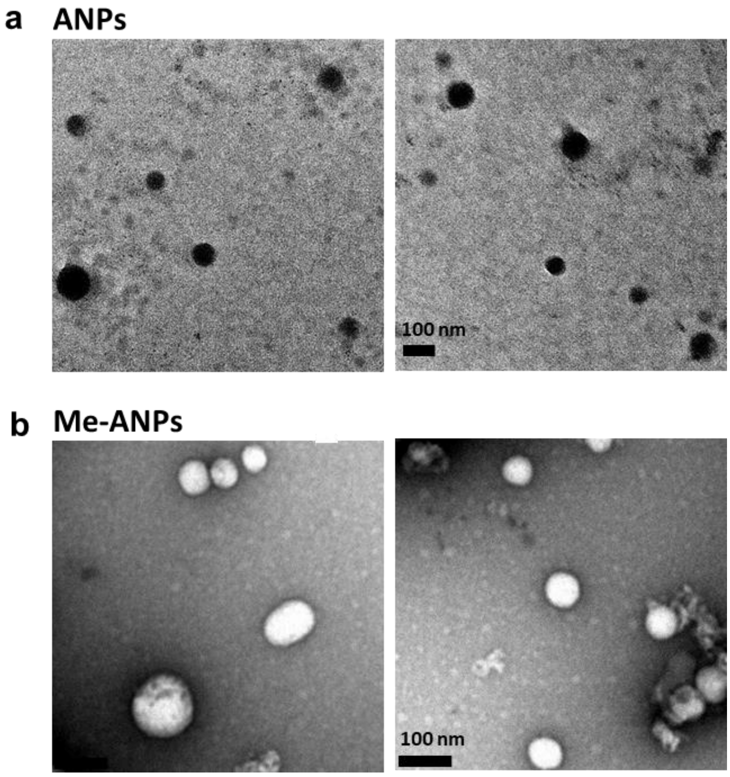

Figure S5. Low-magnification transmission electron micrographs of a negative-stained (0.5% uranyl acetate) (a) ANPs and (b) Me-ANPs after 24 h in 10 mM potassium phosphate buffer at pH 7.4.

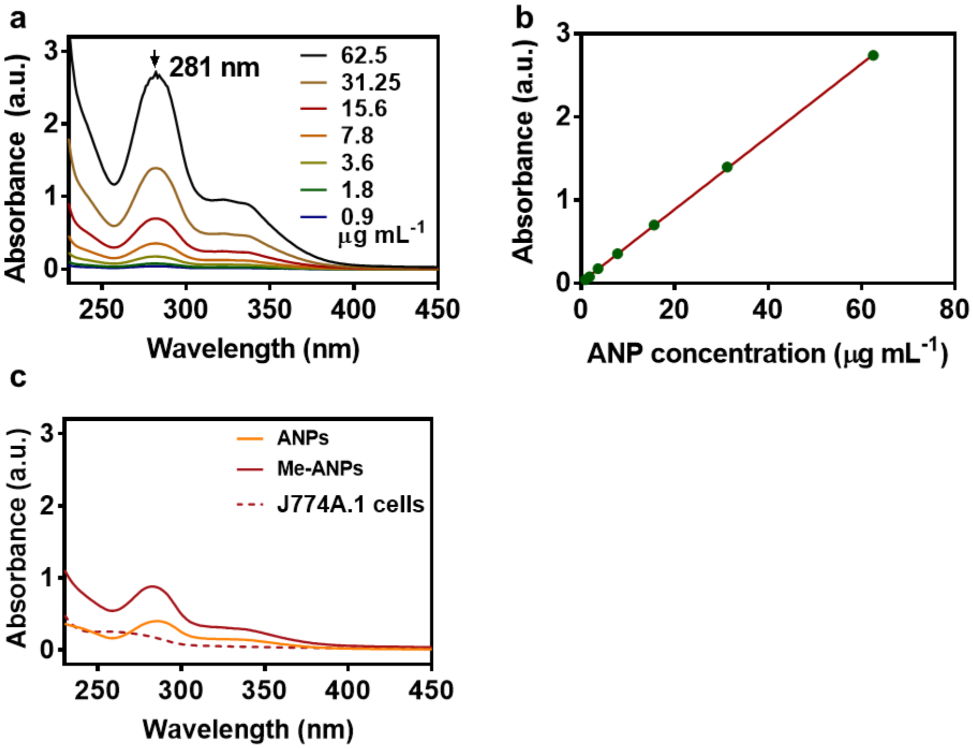

Figure S6. (a) UV-VIS absorption spectra of antimicrobial-conjugated nanoparticles (ANPs) in absence of mouse J774A.1 membrane encapsulation at different conjugate concentrations as a function of wavelength. (b) Linear relation between UV-VIS absorption and antimicrobial-conjugate concentration in a 10 mM phosphate buffer at pH 7.4. (c) UV-VIS as a function of wavelength of antimicrobial NPs in absence and presence of J774A.1 membrane encapsulation (Me) in 10 mM phosphate buffer at 37°C. Spectra for J774A.1 cells only are included as a negative control, confirming absence of absorbance at 282 nm due to antimicrobial-conjugates in cells.

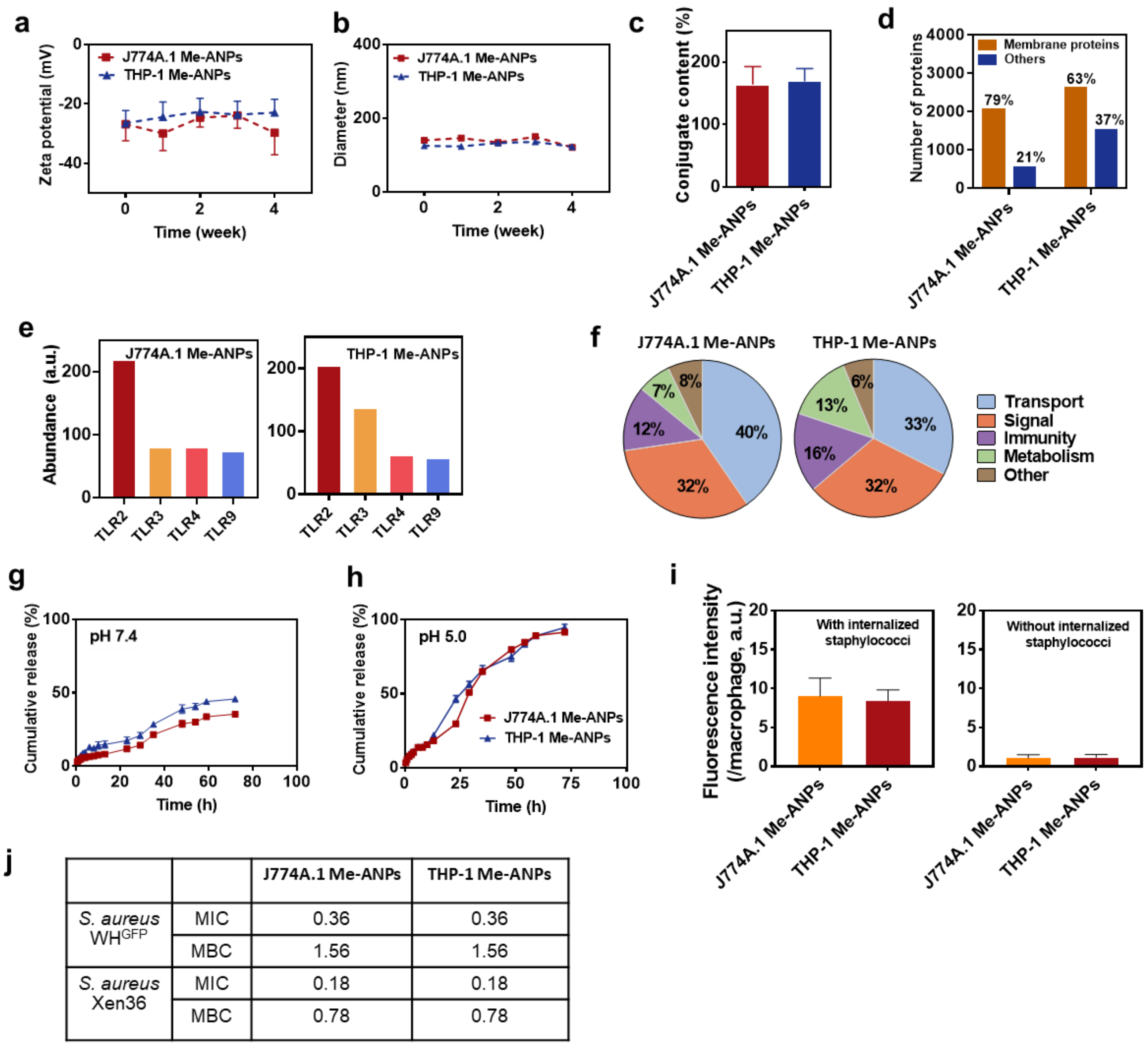

j

		J774A.1 Me-ANPs	THP-1 Me-ANPs
S. aureus WH^{GFP}	MIC	0.36	0.36
	MBC	1.56	1.56
S. aureus Xen36	MIC	0.18	0.18
	MBC	0.78	0.78

Figure S7. Comparison of the properties of mouse J774A.1 and human THP-1 membrane encapsulated, antimicrobial-conjugated ANPs. (a) Zeta potentials (see also Figure 2h), (b) Hydrodynamic diameter (see also Figure 2b), (c) Antimicrobial-conjugate content (see also Figure 2i), (d) The number of membrane proteins and other proteins (see also Figure 2c), (e) Relative abundance of Toll-like receptors (see also Figure 2e), (f) Functional characterization of membrane proteins identified on Me-ANPs (see also Figure 2f). Proteins were classified according to UniProt/GO database, (g) Cumulative antimicrobial-conjugate release at pH 7.4 (see also Figure 3), (h) Cumulative antimicrobial-conjugate release at pH 5.0 (see also Figure 3), (i) Red-fluorescence intensity of Nile red-loaded, red-fluorescent macrophage-monocyte membrane-encapsulated, antimicrobial-conjugated ANPs after entry in sterile and infected, mouse J774A.1 cells without or with intra-cellular *S. aureus* (see also Figure 4b), (j) Minimal

inhibitory and bactericidal concentrations (MIC and MBC, respectively) in μg mL^{-1} of planktonic *S. aureus* WH^{GFP} and *S. aureus* Xen36 in suspension for J774A.1 and THP-1 Me-ANPs (see also Figure 6a). Data are expressed as means ± standard deviations over triplicate nanocarrier preparations.

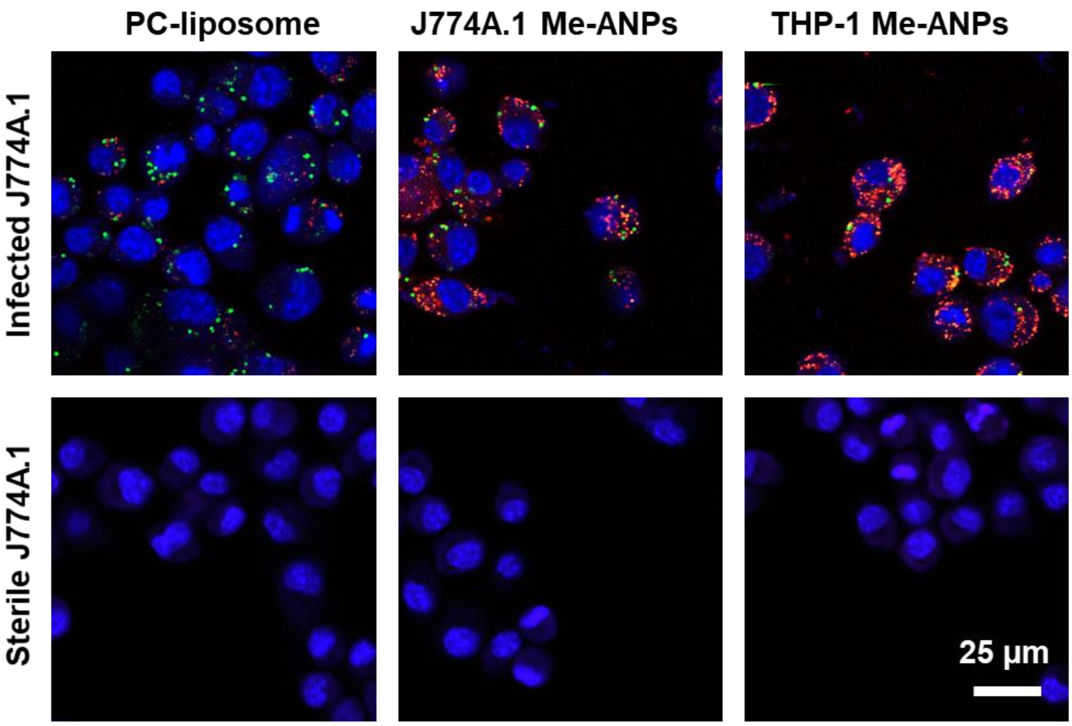

Figure S8. Low magnification CLSM images illustrating intracellular presence of green-fluorescent staphylococci with attached Nile red-loaded, membrane or liposome encapsulated ANPs into J774A.1 macrophages with or without intracellular *S. aureus*. J774A.1 nuclei were blue-fluorescently stained using DAPI.

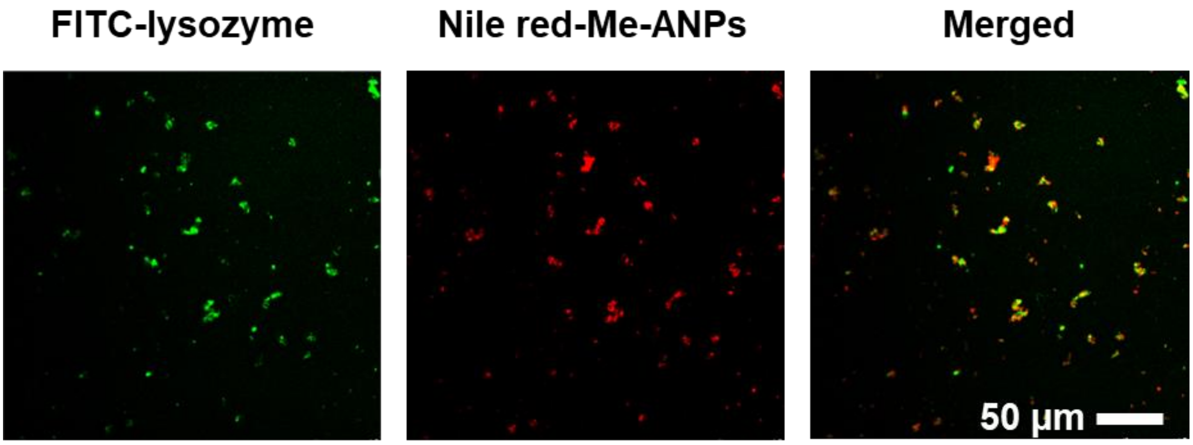

Figure S9. Interaction between lysozyme and Me-ANPs. CLSM images of green-fluorescent FITC-labeled lysozyme and Nile-red loaded Me-ANPs after 12 h incubation, demonstrating co-localization and aggregation of lysozymes and Me-ANPs through electrostatic double-layer attraction.

Method applied: To visualize interaction between lysozyme and Me-ANPs, lysozyme was labeled with fluorescein isothiocyanate (FITC). 20 mg Lysozyme (J&K Chemicals, China) was dissolved in 3 mL 0.1 M borate buffer (pH 9.0) and 1 mg FITC (J&K Chemicals, China) was dissolved in 1 mL DMF. The FITC solution was added dropwise to the lysozyme solution, followed by overnight incubation at 4°C. Next, the mixture was dialyzed against PBS (10 mM, at pH 7.6) and acetate buffer (10 mM, at pH 5.5) to remove residual FITC. Nile red-loaded J774A.1 membrane encapsulated ANPs were prepared as described above. To study the interaction, FITC-labeled lysozyme and Nile red-loaded Me-ANPs were mixed in PBS with the concentration of 0.1 mg mL^{-1} FITC-labeled lysozyme and 0.1 mg mL^{-1} Nile red-loaded Me-ANPs. The mixture was incubated at 37°C for 12 h, followed by centrifugation at 20,000*g* at 4°C for 30 min to collect precipitates. The precipitates were re-suspended in PBS to a final concentration of 1 mg mL^{-1} and CLSM images were taken. Fluorescence signals were collected between 500-535 nm (green-fluorescent FITC in lysozymes) and 583-688 nm (red-fluorescent Nile-red in Me-ANPs).

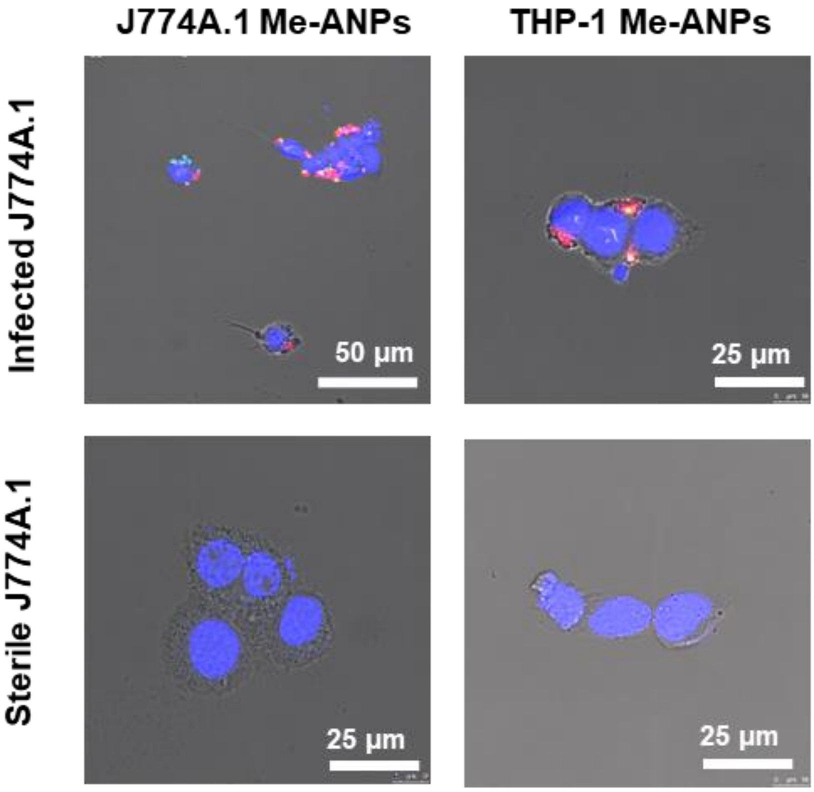

Figure S10. Low magnification CLSM images, illustrating the presence of red-fluorescent lysozyme on sterile and infected J774A.1 cells. Macrophages with or without intracellular *S. aureus* were blue-fluorescently stained using DAPI.

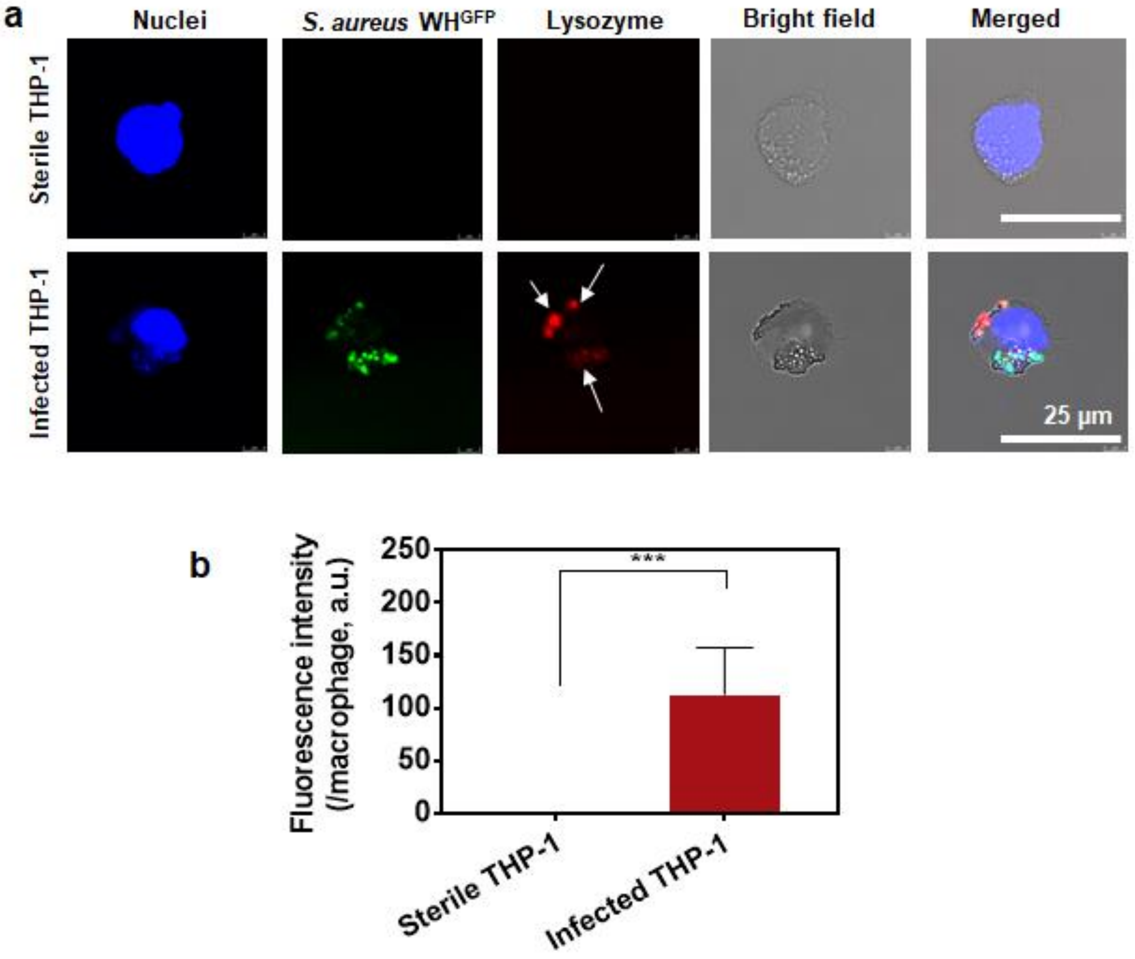

Figure S11. Lysozyme presence on sterile and infected THP-1 cells immediately after infection obtained using CLSM. (a) Demonstration of red-fluorescent, lysozyme-rich scars on human THP-1 cells after infection with green-fluorescent *S. aureus* WH^{GFP}, including absence of lysozyme-rich scars in sterile cells. Blue-fluorescence is due to DAPI staining of macrophage nuclei. Arrows point to lysozyme-rich spots, coinciding with the likely engulfment point of staphylococci in an infected cell. (b) Red-fluorescence intensity due to lysozyme in sterile and infected THP-1 cells measured from the intensity in CLSM images. Data were expressed as a mean fluorescence intensity per macrophage ± standard deviations over triplicate nanocarrier preparations. Three images were blindly chosen in each experiment, comprising on average approximately 5 macrophage per image. Asterisks above the data points indicate statistical significance at $p < 0.001$ (***, Student's *t*-test).

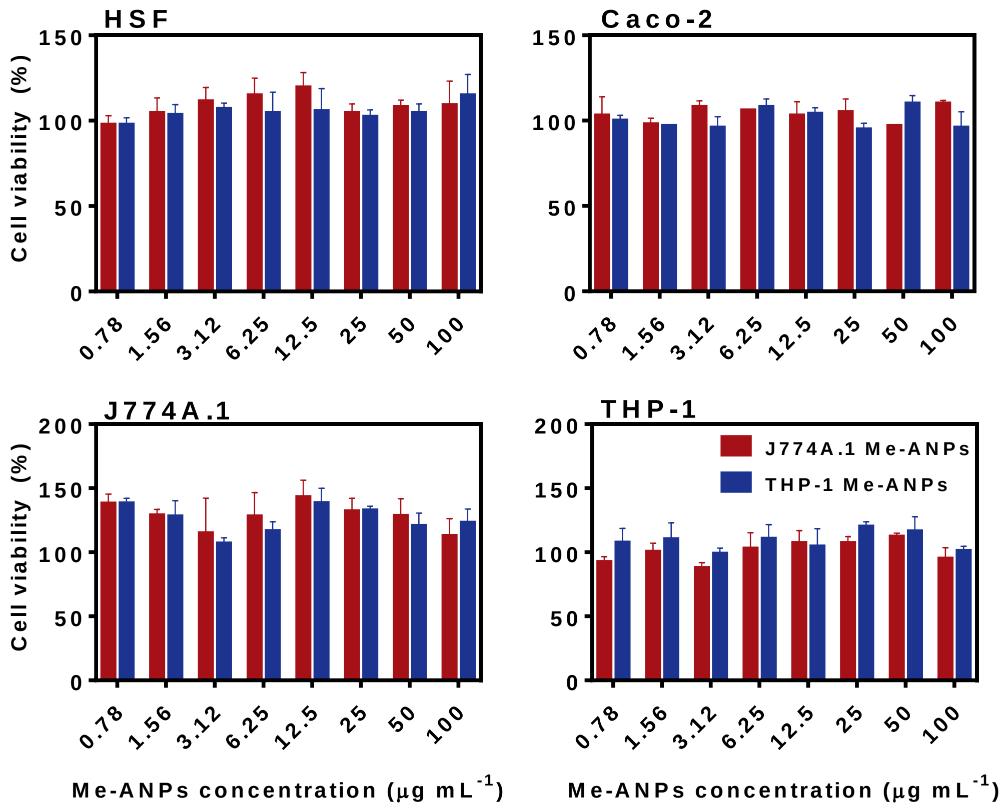

Figure S12. Cytotoxicity of mouse J774A.1 Me-ANPs with different mammalian cell lines. Relative metabolic activity, expressed as percentage MTT conversion of human skin fibroblasts (HSF), human epithelial colorectal adenocarcinoma cells (Caco-2), J774A.1 and THP-1 cells after 24 h incubation in the presence of Me-ANP at different concentrations. MTT conversion in absence of Me-ANPs was set at 100%. All data are expressed as means ± SD values over triplicate experiments with separately prepared micelles and independent cell cultures.

Method applied: Cells (5×10^4 cell mL^{-1}) were seeded into gelatin-coated 96-well plates and incubated at 37°C under 5% CO_2 for 24 h, after which the supernatant was removed. Next, 100 μL suspensions with different concentrations of Me-ANP (0.78-100 μg mL^{-1}) in growth medium were added to each well for 24 h incubation. Subsequently, growth medium was replaced by 100 μL MTT (Sigma-Aldrich, 1 mg mL^{-1}) in growth medium. After 4 h, supernatant was removed and 100 μL i-propanol was added to dissolve formazan and absorbance was measured at 560 nm on a Fluostar® microplate reader (Ortenberg, Germany).

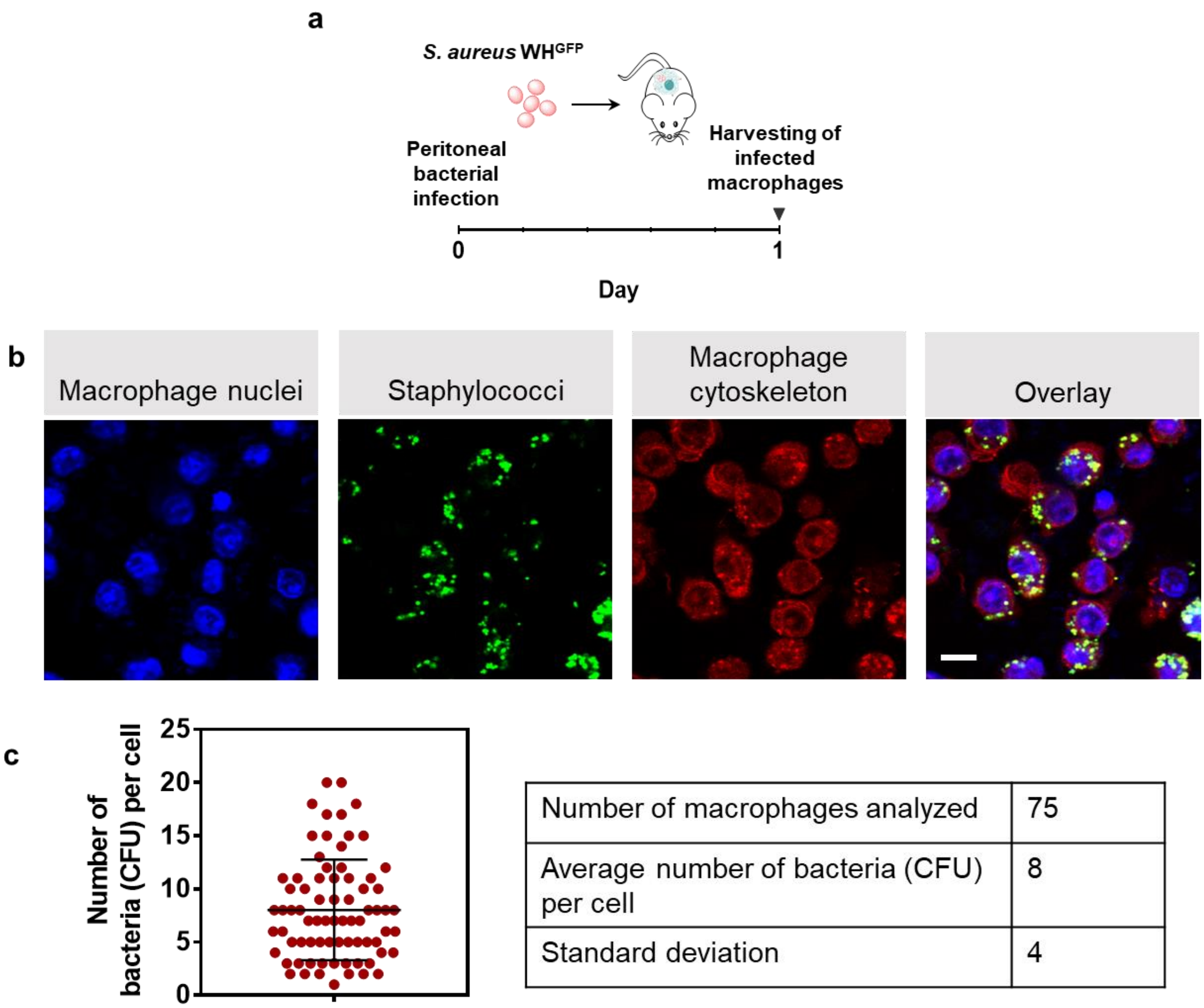

Number of macrophages analyzed	75
Average number of bacteria (CFU) per cell	8
Standard deviation	4

Figure S13. *In vivo* internalization of green-fluorescent *S. aureus* WH^{GFP} in mouse peritoneal macrophages. (a) Schematics of the mouse, peritoneal *in vivo* internalization applied. Staphylococci were internalized after intraperitoneal injection of a staphylococcal suspension and subsequent harvesting of infected, peritoneal macrophages. (b) Examples of CLSM micrographs of *in vivo* internalized staphylococci in macrophages in peritoneal fluid. Scale bar equals 25 μm. Macrophages were stained with blue-fluorescent DAPI (nuclei) and red-fluorescent phalloidin (cytoskeleton). Different fluorescence images were overlaid for quantification of the number of staphylococci per macrophage. (c) Number of staphylococcal CFU per infected macrophage quantified over 75 macrophages taken from peritoneal fluid of three different mice, showing on average 8 staphylococci internalized per macrophage.

Method applied: Macrophage internalization of staphylococci in the mouse peritoneum. Eight-week-old female mice, ICR (CD-1) (35 g to 40 g each) were obtained from Vital River Laboratory Animal Technology Co. (Beijing, China). All animals were housed in the on-site animal facility of Nankai University and experimental procedures were approved by the Institutional Animal Care and User Committee of Nankai University, Tianjin, China. For intraperitoneal infection, each mouse was subjected to intraperitoneal injection (200 μL, 2 × 10^8 CFU mL^{-1}) of a *S. aureus* WH^{GFP} suspension in PBS. At day 1 after infection, peritoneal fluid extract was taken and centrifuged for 5 min at 1500 rpm at 4°C in a table-top centrifuge (5424 R, Eppendorf, Hamburg, Germany). The pellet containing peritoneal macrophages was collected and macrophages were exposed to 50 μg mL^{-1} of lysostaphin[1] (Sigma Aldrich, from *Staphylococcus staphylolyticus*) for 20 min at 37°C to eradicate possible extracellular staphylococci. Next, peritoneal macrophages were washed three times with ice-cold PBS to remove the lysostaphin. This procedure harvested 5 × 10^7 macrophages mL^{-1}. Next, macrophages were lysed and intracellular staphylococci were enumerated after agar plating of serial dilutions, all as described above for *in vitro* internalization.
